# Phylogeny of waterfowl (Anseriformes) constructed using genome sequences provides insights into topological incongruences

**DOI:** 10.1093/molbev/msag018

**Published:** 2026-01-21

**Authors:** Gang Wang, Tao Zhu, Xinye Zhang, Xufang Ren, Anqi Chen, Zhonghua Ning, Marcel van Tuinen, Lujiang Qu

**Affiliations:** State Key Laboratory of Animal Biotech Breeding, College of Animal Science and Technology, China Agricultural University, Beijing, China; State Key Laboratory of Animal Biotech Breeding, College of Animal Science and Technology, China Agricultural University, Beijing, China; Pearl River Fisheries Research Institute (PRFRI), Chinese Academy of Fishery Sciences (CAFS), Guangzhou 510380, China; State Key Laboratory of Animal Biotech Breeding, College of Animal Science and Technology, China Agricultural University, Beijing, China; State Key Laboratory of Animal Biotech Breeding, College of Animal Science and Technology, China Agricultural University, Beijing, China; State Key Laboratory of Animal Biotech Breeding, College of Animal Science and Technology, China Agricultural University, Beijing, China; State Key Laboratory of Animal Biotech Breeding, College of Animal Science and Technology, China Agricultural University, Beijing, China; Department of Biology and Marine Biology, University of North Carolina Wilmington, Wilmington, NC 28403, USA; Biodiversity Hotspots Group, Naturalis Biodiversity Center, Leiden 2333 CR, The Netherlands; Groningen Institute of Archaeology, University of Groningen, Groningen 9712 ER, The Netherlands; State Key Laboratory of Animal Biotech Breeding, College of Animal Science and Technology, China Agricultural University, Beijing, China

**Keywords:** Anseriformes (waterfowl), phylogeny, whole-genome alignment, gene tree discordance, incomplete lineage sorting (ILS), introgression

## Abstract

The evolutionary history of waterfowl (Anseriformes) has long been a focal point of avian research. However, previous phylogenetic investigations have focused primarily on morphology or mitochondrial DNA or have lacked sufficient taxon sampling. Accompanied by observed phylogenetic incongruence and incomplete resolution, waterfowl phylogenetic branching patterns remain uncertain at various taxonomic ranks. To further validate phylogenetic relationships among higher waterfowl taxa and assess presence of conflicting signal, we assembled and analyzed 24 waterfowl genomes representing all waterfowl families and several subfamilies. Utilizing both newly acquired and previously obtained genomes, we constructed and analyzed seven DNA data classes, which yielded highly resolved phylogenetic trees including a time-calibrated tree. Most of these trees consistently and completely resolved the phylogenetic relationships of the included waterfowl species. Despite these efforts, our analysis across chromosomes uncovered four instances of phylogenetic incongruous signal. After minimizing tree estimation error through focus on whole-genome alignment dataset and by sequence simulation, analyses revealed that incomplete lineage sorting and gene introgression essentially contributed to all gene-tree discordance. The variable impact of both factors across distinct waterfowl nodes reflects an underlying complexity that warrants further interpretation. This study not only presents a strongly-supported and well-resolved phylogenetic backbone for the major waterfowl lineages, but also provides foundational data for subsequent comparative genomics studies of a more expanded set of waterfowl taxa.

## Introduction

Waterfowl (Anseriformes), as a branch of the Galloanserae, constitutes a large bird group comprised of more than 180 species, including ducks, geese, swans, screamers, and the magpie goose (Kear 2005). Most modern species in the order are highly adapted to an aquatic existence at the water surface. The majority of available data concerning waterfowl phylogeny has been derived from morphological, anatomical, behavioral, and mitochondrial sequences analyses (Livezey 1997; Sorenson et al. 1999; Donne-Goussé et al. 2002; Gonzalez et al. 2009; Sun et al. 2017). With modern phylogenomic studies continuing to increase representation of bird taxa, phylogenetic resolution has been enhanced across the avian tree along with important insights into sources of topological incongruence. However, while deeper phylogenomic patterns are becoming clearer, the impact on waterfowl phylogenetic resolution has not been discussed. Obviously, scarce sampling of waterfowl (*n* = 1–8) has played an important role here too (Jarvis et al. 2014; Prum et al. 2015; Kuhl et al. 2021; Stiller et al. 2024). Perhaps as a result, existing taxonomic lists of the birds of the world (Lepage et al. 2014), which themselves historically differ somewhat, all continue to divide Anseriformes into three families: Anhimidae (2 genera, 3 species), Anseranatidae (1 genus, 1 species), and Anatidae (57 genera, 173 species) (Livezey 1991, 1995, 1997), and refer Whistling ducks to subfamily Dendrocygninae within Anatidae. Recent analyses based on complete mitochondrial genomes suggest that Whistling duck species should be considered as a separate family, Dendrocygnidae (Sun et al. 2017). This result is in accordance with early genomic attempts at phylogenetic reconstruction through the use of DNA–DNA hybridization data (Sibley and Ahlquist 1990). Monophyly of Anserinae and Anatinae has not formally been investigated with genomic analysis, due to limited efforts in sequencing of diverse waterfowl (excepting geese: Ottenburghs et al. 2016). Therefore, current phylogenetic relationships among the wider “duck,” “swan,” and “geese” genera remain based primarily on insights from morphological and mitochondrial studies.

However, studies in organisms as diverse as primates, Drosophila, yeast, and arthropods have demonstrated that relying solely on morphological or mitochondrial sequences can be insufficient to fully elucidate the phylogenetic relationships among species (Berbee et al. 2000; Satta et al. 2000; Rokas et al. 2003; Murphy et al. 2004; Livezey and Zusi 2007; Rokas and Chatzimanolis 2008). While the phylogenetic and taxonomic placement of Whistling ducks is debated and may require revision, it is but one example of potential phylogenetic uncertainty concerning Anseriformes: e.g. monophyly of an Anhimidae-Anseranatidae clade, phylogenetic placement of *Coscoroba* “swan,” relationships among ecologically disparate ducks, including shelduck (*Tadorna* sp.), perching duck (*Aix/Cairina* sp.), diving duck (*Aythya* sp.), steamer duck (*Tachyeres* sp.), dabbling duck (*Anas/Spatula* sp.), stiff-tailed duck (*Oxyura* sp.), and their relationship to geese and swan.

With the emergence of whole-genome assembly and comparative genome analysis in phylogenetics, it has become feasible to obtain more robust phylogenetic relationships between species and shed light on the underlying causes of conflicting branches (Hackett et al. 2008; Nabholz et al. 2011; Jarvis et al. 2014; Liu et al. 2015; Prum et al. 2015). However, the successful reconstruction of the Tree of Life is subject to multiple factors, such as optimal taxon sampling, sufficient coverage of different categories of genomic regions that vary in substitution rate, selective pressures, and phylogenetic informativeness, and the use of appropriate tree-building models (Reddy et al. 2017; Haag et al. 2022). Once pedigree inconsistencies are minimized to the greatest extent possible, one can truly delve into the biological factors responsible for topological conflicts, such as incomplete lineage sequencing (ILS) and gene flow (or introgression) (Shen et al. 2017; Cai et al. 2021; Zhou et al. 2022). Considerable progress in this regard has been shown recently in the phylogeny of primates, ruminants, and birds (Chen et al. 2019; Shao et al. 2023; Stiller et al. 2024).

In this study, we collected 17 published waterfowl genomes and performed genome assembly and annotation for 7 waterfowl species, which together represent 18 genera across all families of waterfowl. Newly acquired genomes were obtained from genomically unexplored, phylogenetically and ecologically diverse waterfowl species. The genomic data set used to construct the waterfowl phylogeny comprise seven different classes of DNA datasets—coding sequences (CDS), first and second codon positions (codon1&2), third codon positions (codon3), 4-fold degenerate (4d) sites, conserved nonexonic elements (CNEE), whole-genome alignments (WGAs), and Z sex chromosome (ChrZ), with a total of 213.76 MB aligned base pairs. We integrated multiple phylogenomic approaches (including multispecies coalescent models and concatenation) and algorithms (both neighbor-joining [NJ] and maximum likelihood [ML]) to clarify phylogenetic relationships (Jeffroy et al. 2006; Song et al. 2012; Shen et al. 2021). Our findings revealed a fully resolved waterfowl phylogenetic tree, along with phylogenetic conflicts among genomic data classes, and we endeavored to elucidate the reasons for these conflicts.

## Results

### Genome sequencing, assembly and annotation

We used Illumina sequencing technology to generate more than ∼69 Gbase pairs of raw data and then de novo assembled genomes for seven waterfowl species. Genomes were all assembled into large scaffolds with a series of mate-paired short insert libraries and qualified for most comparative genomic analyses. In addition, we downloaded the genomes of 17 waterfowl from public databases and used the Chicken (*Gallus gallus*) genomes as outgroups.

To evaluate the quality of these genome assemblies and downloaded genomes, we performed BUSCO analyses and observed high BUSCO scores (average 96.57%, from 86.90% to 99.06%) (Fig. S1a), showing that the majority of the assemblies were of high quality for downstream comparative analyses. The average length and GC percentage of the newly assembled genomes were 1.1 Gbp and 41.2%, respectively, and all BUSCO scores exceeded 95%. Using a combination of homology and de novo approaches, we found that the proportion of transposable element (TE) sequences in the newly assembled genomes ranged from 11.86% to 17.50%. Long interspersed nuclear elements were the most common repeat type that comprised 6% of the genome. Waterfowl appear to have overall lower TE prevalence than chicken (Fig. S1b).

After masking repeats, we used both de novo and homology-based gene prediction to annotate the genomes. The final annotated gene numbers range from 14,689 to 15,244 among different species, with variations driven primarily by the quality of the assembled genomes. Due to the absence of transcriptome sequences, the completeness of the newly assembled genome annotation was about 60.58% (from 58.72% to 62.44%) (Fig. S1b). The gene length and exon length distributions of newly assembled genomes were similar to mallard duck (GCA_002743455.1) (Fig. S1d and e). We also identified 358,614 waterfowl-specific CNEEs by comparing all the waterfowl genomes to the chicken genome.

### Resolving the waterfowl phylogeny

#### Effect of DNA classes on tree inference

Our genomic data comprise seven different classes of DNA datasets—CDS, first and second codon positions (codon1&2), third codon positions (codon3), 4-fold degenerate (4d) sites, CNEE, WGAs, and Z sex chromosome (ChrZ). We performed intensive sensitivity analysis using both NJ and ML to examine the phylogenomic utility of each of the seven DNA classes on species tree inference (Figs S2–S8).

**Figure 1:**
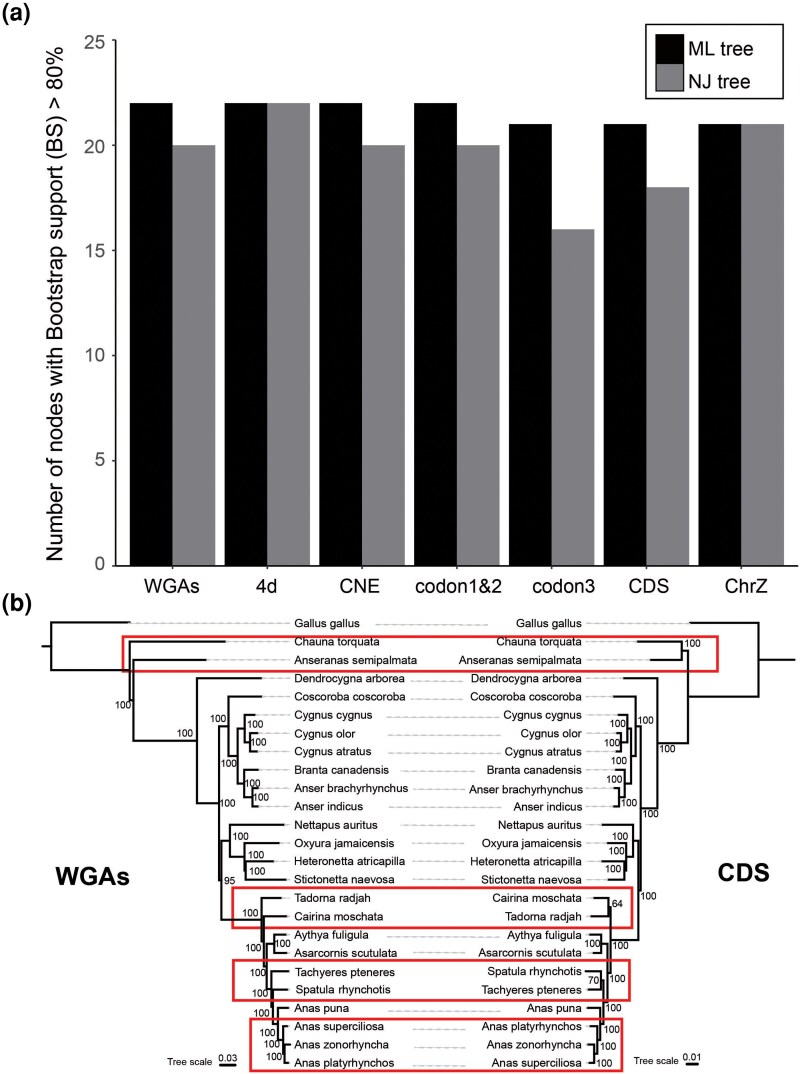
Effects of the individual DNA classes and algorithms on species tree inference and the species tree of waterfowl. (a) Comparison of the number of significantly consensus nodes (BS > 80%) among 22 nodes for trees estimated using NJ and ML algorithms across all seven pairwise combinations of DNA classes. (b) Whole-genome alignments (WGAs) tree constructed using ML method (left) and concatenation coding sequence (CDS) tree constructed using ML method (right). The red box in the figure shows the branches where topological inconsistency exists.

The NJst trees based on the seven individual DNA classes included 4, 2, 6, 0, 2, 2, and 1 low-confidence nodes (bootstrap support [BS] < 80%) with a minimum bootstrap confidence of 73%, 69%, 63%, 98%, 56%, 76%, and 61% for CDS, codon1&2, codon3, 4d, CNE, WGAs, and ChrZ, respectively. Some of these low-confidence nodes were located deep within Anseriformes, indicating that the NJ gene trees built from each of the seven DNA classes alone do not contain sufficient phylogenetic signal to fully resolve the relationships among these waterfowl (Fig. 1a).

Applying a concatenation approach using ML to each of the seven DNA classes produced trees with higher node support compared with corresponding NJ trees. The seven concatenation trees based on individual DNA classes comprised 1, 0, 1, 0, 0, 2, and 1 low-confidence nodes (BS < 80%) with a minimum bootstrap confidence of 64%, 95%, 54%, 82%, 100%, 95%, and 56% for CDS, codon1&2, codon3, 4d, CNE, WGAs, and ChrZ, respectively (Fig. 1a).

The binomial test for differences between the evolutionary trees constructed in the NJst and RAxML methods revealed that comparisons of concatenated ML trees based on different DNA classes resulted in a much lower number of significantly discordant nodes than comparisons of coalescent NJ trees, also underscoring the substantial conflict in phylogenetic signal among the seven DNA classes (Figs 1a and S9). Therefore, we determined that the ML method was more suitable for constructing a robust waterfowl species and analyzing conflicting nodes in waterfowl based on seven different classes of DNA datasets. For comparison to DNA datasets, construction of phylogenetic topology based on protein sequences using the ML method showed the same topology as codon3 (Fig. S10).

#### Whole-genome data reveal comprehensive waterfowl species tree

In total, 47.1 Mb of gap-free data for orthologous syntenic sequences were obtained from WGAs with chicken as a reference genome and used to infer the waterfowl phylogeny by ExaML under the GTR + GAMMA model, yielding a high-resolution whole-genome nucleotide evidence tree (Kozlov et al. 2015). The ML tree has high BS (>95%) for all nodes (Fig. 1b). The coalescent-based consensus tree based on 100 kb non-overlapping windows WGAs yielded the same topology as the ML tree.

The WGAs tree shows that Anhimidae, represented by the Southern Screamer (*Chauna torquata*), is the basal branch of the Anseriformes. The Anseranatidae is composed of its only extant species, the magpie goose (*Anseranas semipalmata*), and is the second-most basal branch of Anseriformes. This tree does not support monophyly of an Anhimidae-Anseranatidae group. *Dendrocygna arborea* represents an independent clade relatively far removed from the remainder of waterfowl, rather than as a genus branching closely within Anatidae. Remaining taxa are divided into two main clades, broadly known as Anatinae and Anserinae (Donne-Goussé et al. 2002). WGAs tree showed that *Coscoroba*, *Anser*, *Branta*, and *Cygnus* form a clade, the traditional Anserinae. *Nettapus* is often considered a unique relic of early duck evolution. In this study, *Nettapus auritus* was placed at the base of the first branch of Anatinae in phylogenetic trees that also includes *Heteronetta*, *Stictonetta* and *Oxyura*. All phylogenetic trees support *Stictonetta naevosa* and *Heteronetta atricapilla* as sister-taxa. Five of the seven species with new genomes in this study are all located in the traditionally more restricted Anatinae, which excludes pygmy geese (*Nettapus*) and stiff-tails (*Oxyura*). This unnamed grouping of restricted anatine waterfowl in this study is composed of four clades. *Tadorna radjah*, representing shelducks, is located in the first clade as earliest branch. *Cairina moschata,* representing perching ducks, forms a second clade. *Aythya fuligula*, representing diving ducks, makes up the third clade together with *Asarcornis scutulata*, a species previously considered a perching duck. Finally, *Anas, Spatula, and Tachyeres*, representing dabbling ducks, are in the fourth most recently diverging clade.

About one-third of all waterfowl belong to the tribe *Anatini*, and many species are conventionally placed in the genus *Anas* (Sorenson et al. 1999). The Mallard complex (*Anas platyrhynchos, Anas superciliosa, Anas rubripes, Anas fulvigula, Anas diazi,* and likely also *Anas zonorhyncha)*, used to be considered a particular problem, with potential hybridization issues equaling those of the large gulls (McCracken et al. 2001; Kulikova et al. 2004; Lavretsky et al. 2014; Sonsthagen et al. 2016). The WGAs tree shows phylogenetic relationships among Anatini with extremely high nodal support. The Australasian shoveler (*Spatula rhynchotis*) was once called *Anas rhynchotis* and considered a member of the genus *Anas*. Mallard (*A. platyrhynchos*) and spot-billed duck (*A. zonorhyncha)* are sister branches of the genus *Anas*. Their common ancestor is a sister branch to the Gray Duck (*A. superciliosa*). The South American *Anas puna* represents an earlier-diverging lineage within *Anatini*.

**Figure 2:**
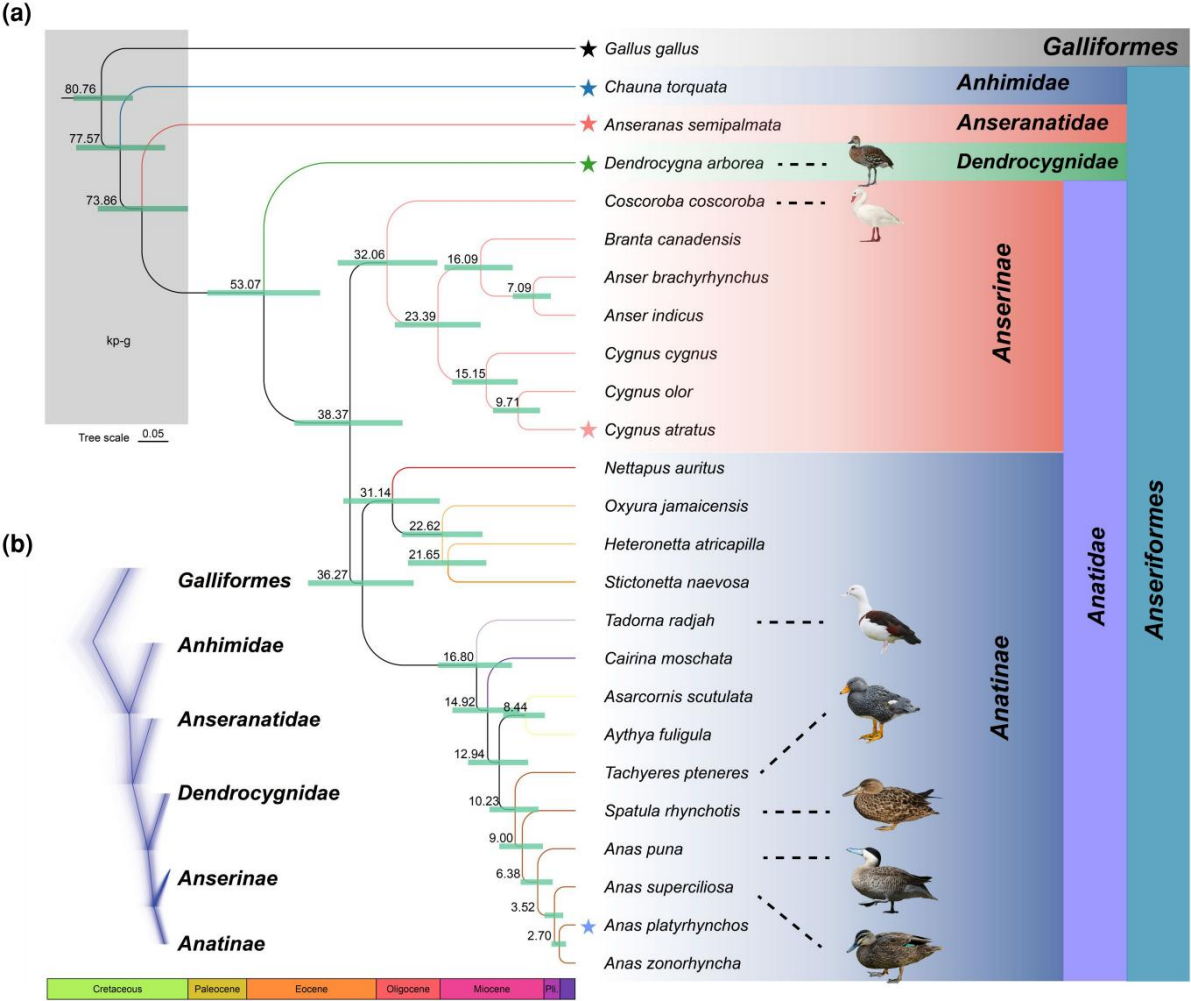
Phylogeny of waterfowl. (a) The maximum likelihood phylogenetic tree from Fourfold degenerate (4d) sites of 24 waterfowl species. The ML tree has the same topology as the phylogenetic tree constructed using WGA. Species marked with stars are representative species of the five major taxa of waterfowl. (b) Prevalent discordance among 5,000 random WGTs was observed across different taxa of waterfowl.

To ensure that the reconstructed subfamily-level branches are robust to the presence of incomplete lineage sorting (ILS), we selected representative species in each phylogenetic subgroup and used DensiTree to analyze 5,000 WGTs. The topology of the obtained tree is the same as that of WGAs trees (Fig. 2b).

### Dating of nodes within the waterfowl tree

The 4-fold degenerate site in the codon does not change the encoded amino acid and is under neutral evolution (Chen et al. 2019). Because the evolutionary tree constructed by concatenating 4-fold degenerate (4d) sites is consistent with the WGAs tree and the CNE tree, it was used for dating analysis with two temporal constraints (Fig. 2a). Recent study shows that among all birds, Neoaves experienced a radiation of species near the Kpg boundary (Claramunt and Cracraft 2015; Wu et al. 2024). Our results show that after Galliformes and Anseriformes diverged in the late Cretaceous, the early-diverging species *Chauna torquata* and *Anseranas semipalmata* in Anseriformes also diverged near the KPg boundary. During the later Pleistocene, climate fluctuations resulted in geographic isolation, habitat fragmentation, and changes in resource availability, which combined to produce tremendous species diversity and richness. These climate fluctuations likely also drove rapid speciation events in waterfowl species (e.g. *Anas, Anser, Cygnus*) (Sun et al. 2017). Furthermore, we not only found that *Dendrocygna* represents an independent clade, but also diverged early from Anatidae at about 53 Mya in the Eocene. These findings advocate that *Dendrocygna* whistling ducks should be classified as a separate family Dendrocygnidae among waterfowl. A second long internode that merits potential taxonomic relevance encompasses the divergence of the first and second branches of traditional Anatinae. The clade of *Nettapus* pygmy geese and *Oxyura* stiff-tail ducks diverged during the early Oligocene while the divergence of remaining strict-group Anatines is more contracted in the Miocene. Whether this pattern warrants taxonomic revision remains unclear, and will require inclusion of sea ducks (tribe Mergini) in future investigation. Sea ducks include merganser (*Mergus*), eider (*Somateria*), scoter (*Melanitta*), and goldeneye (*Bucephala*) ducks, and are generally classified as an early diverging tribe of Anatinae, but this too needs confirmation (Livezey 1995; Sun et al. 2017).

#### Variation in the nucleotide substitution rate

We estimated the overall nucleotide substitution rate in Anseriformes to be ∼0.94 × 10^−9^ substitutions per site per years (Fig. S11), which is much lower than the average rate for birds (∼1.9 × 10^−9^) (Zhang et al. 2014). However, the nucleotide substitution rate exhibited a high degree of heterogeneity between waterfowl lineages, potentially caused by differences with respect to generation time (Fontanillas et al. 2007; Moorjani et al. 2016). Anatinae evolved the fastest at ∼1.14 × 10^−9^ substitutions per site per years. The fastest evolving species among Anatinae is *A. zonorhyncha* (∼1.85 × 10^−9^), whose nucleotide substitution rate is close to the average of birds. Our analysis, in agreement with previous study, suggests that Galloanserae at the base of Neognathae evolved with a slower nucleotide replacement rate compared with other birds (Jarvis et al. 2014).

### Conflicting nodes in waterfowl species trees in different DNA data classes

In the ML trees constructed from 7 DNA data sets, 4d, CNE and ChrZ, completely consistent topological relationships are found with WGA. The trees based on the WGAs coalescent-based consensus and concatenation also have the same topological relationship (Figs 1b and S12). However, CDS, codon1&2, and codon3 differ from WGA in several phylogenetic branching patterns. The phylogenetic positions of the Anhimidae and Anseranatidae families have been strongly debated and this was also the case in our present study when all seven genomic data sets were utilized and compared for phylogenetic purposes. All three exon-based data sets (including protein dataset) show that these two families are sister clades, while WGA, CNE, 4d, and ChrZ shows that Anhimidae, as the base of waterfowl, is an outgroup to other waterfowl. At this node, the support of CDS and codon3 is 100%, while the support of codon1&2 is only 54%. In addition, we obtained the coalescent-based consensus species tree with the dataset of CDS (Fig. S13). The resulting gene tree is also in dispute with the WGA tree at this node. These conflicting tree topologies imply that the incongruence lies primarily in the coding regions which are generally under more selection. The second conflicting node appears for the branching of *Tadorna*/*Radjah* and *Cairina*. CDS and codon3 regard the two as sister clades, but WGAs shows support for basal split of *Tadorna*/*Radjah* and monophyly of the remaining Anatinae with *Cairina*. The CDS tree shows a lower support of 64%. The phylogenetic placement of *Tachyeres* likewise shows considerable conflict. Codon1&2 and CDS show that *Tachyeres* clusters with *Spatula*, but WGA shows that *Tachyeres* is basal to *Spatula* and *Anas*. Here, all trees exhibit extremely high confidence values (BS ≥ 99%) at this node (Fig. 1b). The last conflicting node is in genus *Anas*, particularly the Mallard Duck complex. Species in the mallard complex are known for frequent hybridization among other members of the complex (Lavretsky et al. 2014). All phylogenetic trees constructed using coding regions show that *A. platyrhynchos* is a sister branch to *A. superciliosa*/*A. zonorhyncha*, while WGA, CNE, ChrZ, and 4d show completely different trees. These controversies are probably attributable to ILS and gene introgression in conjunction with the short internal branches in the dabbling duck radiation.

### Relative contributions of ILS, tree estimation error, and gene introgression to gene tree heterogeneity

If the time between two consecutive speciation events is short and/or the effective population size (Ne) is large, then genes from the two most closely related species may coalesce deeper in the past than the time of the oldest speciation event (Mailund et al. 2014). This can result in genealogical histories that are different from the species tree—a phenomenon called ILS (Suh et al. 2015). ILS has affected the evolutionary history reconstructed for primates and birds (Rivas-González et al. 2023; Stiller et al. 2024). Gene introgression and tree estimation errors can also lead to inconsistent gene trees and result in incorrect species tree estimates (Cai et al. 2021). Therefore, we focused next on the effects of tree estimation error, ILS, and gene introgression in constructing waterfowl gene trees and quantified their relative contributions.

The gene concordance factor (gCF) can be used to quantify genealogical concordance and assess the variation among gene trees (Minh et al. 2020). By calculating the gCF of the CDS dataset using IQ-TREE (Minh et al. 2020), we found that the average gCF of each node is only 57.78% (Fig. S14). All nodes were detected with varying degrees of ILS, and introgression was detected for one-third of the nodes. The phylogenetic placement of *Anseranas* showed the strongest introgression signal, and the Mallard complex the highest ILS signal. Relative importance decomposition analysis across all internal nodes of the phylogeny waterfowl revealed that ILS, gene tree inference error, and gene introgression jointly explain 89.15% of the total gene tree heterogeneity using the lmg algorithm (*R*^2^ = 0.8915). ILS, tree estimation error, and gene introgression contributed 77.17%, 17.81%, and 5.02% of the gene tree heterogeneity, respectively (Fig. S15). Thus, for this data set, tree estimation error is also identified as a significant factor in the overall heterogeneity of the gene tree.

**Figure 3:**
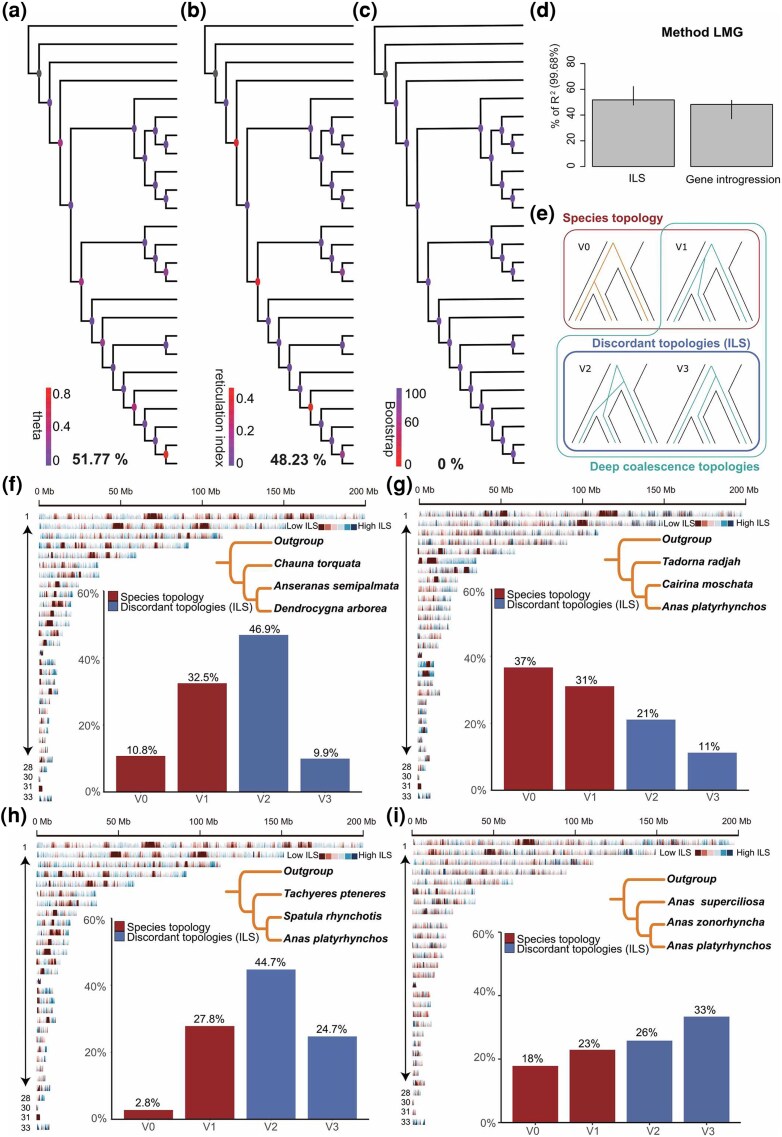
Relative contributions of ILS, gene tree estimation error, and gene introgression across the phylogeny of waterfowl by using the 100 kb non-overlapping windows of WGAs and ILS of four topological incongruences in waterfowl phylogeny. (a) ILS. Nodes are colored by inferred population mutation parameter theta. (b) Gene introgression. Nodes are colored by Reticulation Index. (c) Gene tree estimation error. Nodes are colored by bootstrap (BP) values, which represent percentage of recovered nodes from sequence simulation. (d) The ILS and gene introgression contributed to the heterogeneity of the gene tree were estimated based on LMG regression methods. (e) The four different types of genealogy inferred by CoalHMM. The sum of V2 and V3 topologies is the total percentage ILS. (f) Genome-wide ILS distribution when the original combination of species is (Outgroup (*Chauna torquata* (*Dendrocygna arborea*, *Anseranas semipalmata*))). Horizon plot of the mean *z*-standardized ILS values in 100-kb windows (*x* coordinates in Mb). Red colors represent regions low in ILS, and blue colors represent high-ILS regions. The proportions of support for the four different types of genealogy are shown in the column chart. (g) Genome-wide ILS distribution when the original combination of species is (Outgroup (*Anas platyrhynchos* (*Tadorna radjah*, *Cairina moschata*))). (h) Genome-wide ILS distribution when the original combination of species is (Outgroup (*Anas platyrhynchos* (*Tachyeres pteneres*, *Spatula rhynchotis*))). (i) Genome-wide ILS distribution when the original combination of species is (Outgroup (*Anas superciliosa* (*Anas platyrhynchos*, *Anas zonorhyncha*))).

Compared with the CDS dataset, the 100 kb non-overlapping window WGAs dataset showed higher average gCF (86.37%) (Fig. S14). Furthermore, we detected ILS and gene introgression on the same nodes, with each node fully resolved-indicative of minimal tree estimation error. Interestingly, when using the regression model to decompose the relative importance of gene tree heterogeneity, ILS and gene introgression jointly explained 99.68% (*R*^2^ = 0.9968) of the total tree heterogeneity. ILS was still the most significant factor (51.60%), but gene introgression contributed nearly equally (48.07%) (Figs 3a–d and S16). The higher model prediction ability of gene tree heterogeneity of this data set compared with the single copy gene data set mirrors the gCF results and demonstrates the robustness of WGAs in constructing species trees.

### Causes of topological discordance on a node-by-node basis

In addition to quantifying gene tree heterogeneity, we applied the autoCoalHMM pipeline to analyze four nodes mentioned above using combinations of quartets of species from the genome-wide alignment (Rivas-González et al. 2023). After filtering out ambiguously aligned regions, we used posterior decoding to infer segments of the alignment best supported by either the species topology or any of the two possible discordant topologies. This approach more intuitively displays the degree of ILS in different regions of the genome.

We found an appreciable genome-wide ILS proportion of 56.8% where the inferred relationship of species was (Outgroup (*Chauna torquata* (*Anseranas semipalmata*, *Dendrocygna arborea*))) which implies that, on this quartet, a large proportion of the genome follows a different gene genealogy from that of the species tree. This result is consistent with the observation in the phylogenetic tree that the trees for CDS, codon 1st & 2nd, 3rd, and protein datasets differed from the WGA tree (Figs S5, S6, and S8). In addition, we observe extremely different proportions of genomic positions assigned to the two discordant topologies, with V2 (46.9%) being almost 5.5 times higher than V3 (9.9%) (Fig. 3e and f). Indicative of ancient gene introgression between Anhimidae and Anseranatidae, ABBA-BABA test (also called *D*-statistics) provided further evidence for introgression in their evolutionary history (Chen et al. 2019). No matter which species of Anatidae (P2) was used as representative of the sister group of Anseranatidae (P1), gene introgression could be detected (*D*-statistics >0.1 and *Z*-score >20) (Fig. S17a). Therefore, we hypothesized that the cause of conflicting branches was not only caused by ILS, but that introgression may have also played an important role. Phytop analysis (Shang et al. 2025) verified this assumption. ILS and gene introgression explained 21.5% and 28.2% of the tree topological incongruence, respectively (Fig. S18a).

The second position that is inconsistent in multiple phylogenetic trees contains the original phylogenetic inference of species: (Outgroup (*Tadorna radjah* (*Cairina moschata*, *Anas platyrhynchos*))). The proportion of ILS was 31%, with a nearly one-to-one ratio of V2 (21%) to V3 (11%) (Fig. 3g). Gene introgression analysis showed that *Cairina moschata* and *Tadorna radjah* also had low introgression levels (*D*-statistics >0.05 and Z-score near the threshold) (Fig. S17b). Furthermore, ILS and gene introgression explained 15.7% and 9.1% of the tree topological incongruence, respectively (Fig. S18b). These results indicate that ILS is an important reason for the controversy in the branch, rather than introgression.

Further study on the inferred relationship between species: (Outgroup (*Tachyeres pteneres* (*Spatula rhynchotis*, *Anas platyrhynchos*))) showed the highest ILS (69.4%) among the four incongruent branches (Fig. 3h). No matter which species of the genus *Anas* was used as the sister group of *Spatula rhynchotis*, the value of the *D*-statistic is greater than 0.05 and the *Z*-score is greater than 10 (Fig. S17c). The contribution of gene introgression (30.9%) was greater than that of ILS (24.1%). These results showed that although ILS was an important reason for the conflicting signal at this node, gene introgression between *Tachyeres* and *Spatula* relatively played and even more important role (Fig. S18c).

For the final topological conflict, we focused on the Mallard Complex. The original inferred relationship between species: (Outgroup (*Anas superciliosa* (*Anas platyrhynchos*, *A. zonorhyncha*)) also displayed a high level of ILS (59%) with a nearly one-to-one ratio of V2 (26%) to V3 (33%) (Fig. 3i). Species in the Mallard complex are known for frequent hybridization among other members of the complex. The species within the complex can be very difficult to distinguish genetically, likely due to the retention of ancestral genetic variation. Gene introgression exists in the Mallard complex but the values are relatively low, as evidenced by the close but not equal V2/V3 ratios. The analysis based on phytop showed that ILS contributed a high rate (55.2%) to the tree heterogeneity, while gene introgression contributed a low rate (2.5%) (Fig. S18d). Our results confirm that gene introgression plays a role in the Mallard Complex, but strongly suggest that ancestral genetic variation has obscured the phylogenetics of the Mallard complex even more strongly.

### Genomic dynamics at the base of waterfowl

Utilizing the resolved waterfowl phylogeny by the 4-fold degenerate (4d) sites, we identified rapidly evolving genes (REGs), positively selected genes (PSGs), and instances of gene family expansion and contraction (Figs 2a and S19a). Functional enrichment analysis revealed that REGs along the ancestral waterfowl branch were significantly associated with development of multiple tissues and energy metabolism terms (GO:0090090, GO:0120254, GO:0043434). Furthermore, several immune-related genes (GCH1, TST, PGD, CCL20) were also identified within the PSGs set. Concurrently, we observed a contraction in the MHCY1 major histocompatibility complex gene family. Beyond immune-related genes, we also detected a series of PSGs and REGs involved in response to peptide hormone, enzyme-linked receptor protein signaling pathway, and animal organ development (Tables S2–S6). Compared with Galliformes, waterfowl consistently showed enrichment across gene sets (whether contracting, expanding, rapidly evolving or positively selected) related to muscle physiology and neuromuscular signaling (Fig. S19b). While waterfowl exhibit specialized adaptations for swimming, aquatic foraging and sustained flight, the role of these genes in enhancing such adaptations remain putative and require functional validation.

## Discussion

To date, although research on waterfowl phylogeny has been common, most investigations still rely entirely on outcomes of studies that use morphology, mitochondria or a small number of nuclear genes (sequence length less than 16 kb). This reliance has led to uncertainty and conflicting interpretations of the finer classification of waterfowl. It has been demonstrated in studies of birds and mammals alike that no single gene tree exists identical to the species tree, suggesting that phylogenetic studies based on one or more genes, especially for rapid radiations, may not be sufficient (Edwards et al. 2005; Feng et al. 2022). In this study, we not only collected all waterfowl genomes that have been published so far, but also assembled several new waterfowl genomes. Through genome annotation and whole-genome comparison, we obtained seven distinct (sub)sets of genomic data for each genome, including WGA, and constructed an evolutionary tree of waterfowl using concatenation and coalescent methods. We found that compared with the NJ method, the ML method can obtain higher-resolution evolutionary trees, consistent with results of other bird studies (Wu et al. 2024). Our analyses yielded trees with full statistical support for all deep waterfowl nodes and interspecific nodes using both coalescent and concatenation approaches. Noteworthy, full agreement was found between WGA, 4d, CNE, and ChrZ. Nonetheless, even this comprehensive effort identified phylogenetic conflict among individual gene trees, as well as between gene trees and species tree (Fig. 3).

The WGAs trees constructed by the concatenation and coalescent methods showed that Anhimidae and Anseranatidae, as two families with a relatively small number of species among the three waterfowl families, were the first to differentiate from other waterfowl. Unlike previously published mitochondrial studies and in agreement with recent genomic studies (Stiller et al. 2024), the WGAs trees showed that the two families did not form a monophyletic group, but that Anhimidae diverged first and then Anseranatidae. However, not all seven genomic data types yielded consistent results; exonic data sets (CDS, codon1&2 and, codon3) were in agreement with previous mitochondrial studies (Fig. 1b).

Conversely, the relatively isolated position of *Dendrocygna* between *Anseranas* and other waterfowl, confirms findings from both mitochondrial DNA and earlier genomic efforts (Sibley and Ahlquist 1990; Sun et al. 2017). Our data do not show conflicting topological signal across DNA data classes for this branch. At this point, evidence appears strong to consider upgrading Whistling ducks to family Dendrocygnidae.

The conflicts we do observe between different data types can no longer be considered to be due to error from small amount of sequence data or to differences in concatenation versus coalescent methods (Ericson et al. 2006; Hackett et al. 2008; Kimball et al. 2013). Incomplete lineage sequencing (ILS) and introgression, as biological factors leading to branch conflicts in reconstructed phylogenies, may also be important causes of conflict in waterfowl phylogenies (Mailund et al. 2014; Chen et al. 2019; Feng et al. 2022; Rivas-González et al. 2023). ILS has affected the evolutionary history of the human genome as well as many other groups (Suh et al. 2015; Wang et al. 2018). Around 30% of the human genome does not follow the ([human, chimpanzee], gorilla) speciation tree, with 15% of nucleotide positions grouping human and gorilla, and 15% grouping gorilla and chimpanzee (Scally et al. 2012; Mailund et al. 2014).

The level of ILS generally increases with shorter internal branch lengths. Through the analysis of species divergence times with fossil calibration, we found that the divergence times of Anhimidae and Anseranatidae were very close and near the end of the Late Cretaceous, at a time just before or perhaps coinciding with the large radiation of bird taxa in Neoaves (Stiller et al. 2024; Wu et al. 2024). Genome-wide ILS screening found that the proportion of ILS in this branch reached 56.8%, which strongly suggests that ILS is the main source of incongruence between gene and species trees and between species trees inferred using alternative approaches and datasets (Fig. 3f). Previous studies based on genome-wide phylogenies concluded that reticulate evolution (gene introgression) events are common in branches of the avian phylogenetic tree, especially during the period of evolutionary radiation (Suh et al. 2015). Therefore, evolutionary radiation not only leaves traces of ILS, but also has the potential to leave detectable gene introgression in the genome. To estimate to what extent the phylogenetic incongruence that the model attributes to ILS is affected by widespread gene introgression in this branch, we investigated the relative frequency assigned to the two discordant topologies on the branch. If explained by ILS, this measure should be equal for the two discordant topologies, whereas hybridization is expected to cause one of the discordant topologies to be more frequent (see the proportions of V2 versus V3, Fig. 3). We observed different proportions of genomic positions assigned to the two discordant topologies, suggesting that hybrid introgression is also an important contributor to the observed phylogenetic incongruence among waterfowl. The ABBA test and phytop analysis also provided evidence for gene introgression (Figs S17a and S18a).

Most phylogenetic incongruence between genomic data classes appears in Anatinae. Unlike the phylogenetic placement of *Anseranas*, a lower degree of introgression was detected in the branching of *Tadorna radjah* with respect to *Cairina moschata*. ILS rather than gene introgression is the main reason for the discordance in phylogenetic support. However, if a pair of hybrid species does not leave living descendant species (which may be because most species become extinct), this would not have been distinguishable from deep coalescences in causing ILS. Thus, we cannot rule out the influence of gene introgression as this would not have been distinguishable from deep coalescences—just that it left no or less detectable evidence of ancient hybridization. In contrast to this pattern, we found that the branches leading to *Tachyeres pteneres* and *Spatula rhynchotis* exhibit strong gene introgression. In agreement, a recent study proposed an ancient introgression event from *Tachyeres* steamer ducks into *Anas* dabbling ducks (Zhang et al. 2024). Our results provide new evidence for introgression between *Tachyeres* steamer ducks and *Anas* dabbling ducks (Figs 3h and S18c). The divergence time indicated by the time tree suggests that the two species branched off relative to *Anas* within less than 1.5 Mya from each other. This short divergence period may have contributed to the complex inference of phylogenetic relationship between the two taxa at the whole genomic level, and is likely driven by both ILS and gene introgression resulting from a rapid species radiation. Furthermore, *Tachyeres pteneres*, one of the flightless ducks in *Tachyeres*, and the domesticated species within *Anas*, share a remarkably close phylogenetic relationship. This connection provides valuable insights for future research on the convergent evolution of flight loss in these species. Reticular evolution can increase species diversity and environmental adaptability through adaptive gene introgression, so the detection of gene introgression at multiple nodes also shows that introgression cannot be ruled out as a factor.

In summary, the strongly supported phylogenetic tree based on whole-genome, Z-chromosome, and conserved noncoding element alignments confirms that screamers alone form the oldest living branch of waterfowl, that whistling ducks warrant family status, that “Anserinae” and “Anatinae” are each monophyletic, that the Coscoroba swan is the oldest anserine branch and that perching ducks are not monophyletic. Furthermore, results indicate that pygmy-geese (*Nettapus*) represent an early branch of an anatine clade that also includes freckled (*Stictonetta*) and stiff-tail ducks (Oxyura), that shelduck (*Tadorna*), diving duck (Aythya), and dabbling ducks (Anas/Spatula) are distinct waterfowl branches, and that steamer duck (Tachyeres) is basal to the true dabbling (*Anas*) and shoveler (*Spatula*) ducks. Although our phylogenetic analysis has covered the five major lineages of waterfowl lineages (e.g. *Chloephaga*, *Cereopsis, Plectropterus* “geese,” merginine sea ducks, *Merganetta* river ducks and austral *Chenonetta*, *Hymenolaimus*, *Biziura* ducks) could not be included in this study. Therefore, their phylogenetic position among waterfowl needs confirmation, which will aid in further quantifying genome-level topological incongruence. With the development of The Bird 10,000 Genomes Project (B10K) and the popularity of genome assembly, we hope to conduct more detailed studies on the phylogeny of waterfowl based on whole-genome comparisons in the future (Stiller et al. 2024).

## Materials and methods

### Sample collection

The complete set of waterfowl genomes used in this study contained 24 species, including 17 previously published genomes and 7 newly assembled genomes of *Anas puna*, *Spatula rhynchotis*, *Coscoroba coscoroba*, *Dendrocygna arborea*, *Tachyeres pteneres*, *Tadorna radjah*, and *A. superciliosa*. In addition, we also collected the genome of Chicken (*Gallus gallus*) for outgroup purposes and subsequent whole-genome comparisons (Table S1).

Blood samples of seven waterfowls were collected from individuals bred at the Sylvan Heights Waterfowl Center in North Carolina. All animal specimens were collected legally and in accordance with the policy of the Animal Care and Use ethics of each institution, which meets or exceeds US regulatory standards for the humane care and treatment of animals in research.

### DNA sample preparation and sequencing

Genomic DNA of seven species was extracted from blood samples following the protocol of DNeasy Blood & Tissue kit (Qiagen, USA). A total of 20 μl of proteinase K was added into a 2 ml microcentrifuge tube containing 220 μl of anticoagulated blood. Then, 200 μl of Buffer AL (without added ethanol) was added. The solution was mixed thoroughly by vortexing, and incubated at 56 °C for 10 min. Then, 200 μl of ethanol was added to the sample, and the solution was mixed thoroughly by vortexing. The mixture was pipetted into a DNeasy Mini spin column and centrifuged at 6,000 g (8,000 rpm) for 1 min. The DNeasy Mini spin column was placed in a new 2 ml collection tube, 500 μl of Buffer AW1 was added, and the mixture was centrifuged for 1 min at 6,000 g (8,000 rpm). Next, 500 μl of Buffer AW2 was added to the spin column and centrifuged for 3 min at 20,000 g (14,000 rpm) to dry the DNeasy membrane. Finally, 50 μl of Buffer AE was placed directly onto the DNeasy membrane and centrifuged for 2 min at 6,000 g (8,000 rpm) for elution, after incubation at room temperature for 5 min. DNA aliquot was then precipitated into pellet form. To construct short-insert libraries for Illumina sequencing, 5 µg of DNA was sheared into fragments of 150 bp, end-repaired, A-tailed and ligated to Illumina paired-end adapters (Illumina, San Diego, USA). The double-terminal library of Illumina Hiseq 4000 platform was built with reads of 2 × 150 bp in average length. In total, we obtained ∼69 Gb of raw data. All the genomic sequences were generated by Novogene Inc, Beijing, China.

### Genome assembly and completeness evaluation

From raw data of each species, adaptors were removed, low-quality bases trimmed and “N” sites with fastp (v.0.20.0) (Chen et al. 2018) removed. Cleaned reads were passed through MEGAHIT (v.1.2.9) for de novo assembly (Li et al. 2015). First, MEGAHIT was used to construct a de Bruijn graph by splitting reads of short insert sizes into k-mers, merging k-mers while clipping tips and bubbles, and removing low-coverage links. Then, the contigs which exhibited unambiguous connections in de Bruijn graphs were collected. We tried different k-mers (from 39-mer to 89-mer) to construct contigs and chose the k-mer with the largest N50 contig length with the parameters “–distanceLow 100 –distanceHigh 1,500 –fastMap.” The gap-filling step was performed by AlignGraph with the mallard duck genome (GCA_008746955.3) as reference (Bao et al. 2014). The completeness of the genome after primary correction is still low. Subsequently, Ragtag (v.2.10) was employed to further build scaffolds (Alonge et al. 2022).

The final scaffold genome was evaluated with BUSCO (V.5.7.0) to assess the genome completeness by estimating the percentage of expected single copy conserved orthologs captured in our assemblies, referring to the aves_odb10 BUSCO set (Seppey et al. 2019). The other 17 genomes were also evaluated by the same pipeline. In addition, we assessed the annotation completeness of the newly assembled genomes using BUSCO.

### Genome protein-coding gene annotation

Genomes need to be masked before annotation of protein-coding genes. To annotate the repeat content, we first used RepeatModeler2 to predict and classify TEs throughout the genome (Flynn et al. 2020). The newly predicted families of TEs and tandem repeats were then combined with the Repbase library (http://www.repeatmasker.org/) (RepBase17.01) to annotate repeats using RepeatMasker (v4.0.7) (http://repeatmasker.org) (Tarailo-Graovac and Chen 2009). In addition, we used LTR_finder to identify long terminal repeat (LTR) sequences (Ou and Jiang 2019).

The final assembly's structural annotation of genes was conducted using de novo prediction and homology-based prediction. For de novo gene prediction, we utilized BRAKER2 (v.2.1.6) to analyze the repeat-masked genome with the orthologous protein as hints to generate predicted gene models from AUGUSTUS (v.3.4.0) and to train the hidden Markov model (HMM) of GeneMark-ET (v.3.67_lic) (Gabriel et al. 2021). Protein sequences of human (GCA_000001405.29), chicken (GCA_016699485.1) and mallard duck (GCA_008746955.3) were used as templates for homology-based gene prediction for all of the newly assembled genomes by Miniport (v.0.13) (Li, 2023). EVidenceModeler software (EVM, v.1.1.1) was used to integrate the genes predicted by the homology and de novo approaches and generate a consensus gene set (Haas et al. 2008). Short-length (<50 aa) and prematurely terminating genes were removed from the consensus gene set, and the final gene set was produced.

### Identification of CDS and signatures of genomic dynamics at the base of waterfowl

To identify orthologous CDS sequences across species, we applied the protein sequences of the chicken as a query against the protein sequences of the remaining 24 species using the program OrthoFinder (v2.3.12) with an *E*-value cutoff of 1e^−10^ (Emms and Kelly 2019). A total of 0.53 Mb of single-copy orthologous protein sequences were obtained. Subsequently, the ML tree was constructed using IQ-TREE under the automatic model selection mode (-m MFP), which employs ModelFinder to determine the best-fit substitution model based on the Bayesian Information Criterion (BIC) or Akaike Information Criterion (AIC) (Minh et al. 2020). Then, the script pal2nal.pl was used to convert the single-copy orthologous sequences into CDS nucleotide sequence (Suyama et al. 2006). Finally, the sequences obtained after concatenation were aligned by using MAFFT (v.7.505) (Katoh and Standley 2014). We then filtered out those candidate orthologs less than 100 bp in sequence length. As a result, a total of 1.62Mb CDS sequences were retained for further analysis. In CAFE5 (v.5.0), a random birth-and-death model is used to study expansion and contraction in gene families across a user-specified divergence time tree, which was obtained using MCMCTREE on a 4d locus DNA data class as shown in the subsequent Divergence time calibration analysis (Fig. 2a) (Yang 2007; Mendes et al. 2021). In the results, we only present gene family expansion and contraction on the ancestral branch of waterfowl.

Nonsynonymous and synonymous substitution rates (Ka/Ks) were calculated using the codeml program in the PAML (v.4.5) package (Yang 2007). Since the codeml program requires an unrooted tree as input, the tree topology shown in Fig. 2a was used as the prior tree topology, and the “ape” package was used to estimate the unrooted tree topology for each orthologous gene. Positive selection signals on genes (PSGs) along waterfowl lineage were detected using the optimized branch-site model. A likelihood ratio test (LRT) with df = 2 was conducted to compare a model that allowed sites to be under positive selection on the foreground branch (ancestral branch of waterfowl) with the null model in which sites could evolve either neutrally and under purifying selection. The *P*-values were computed based on Chi-square statistics, and genes with *P* value less than 0.05 indicate a significantly better fit by the alternative model. *P*-values were adjusted for multiple testing using the Benjamini–Hochberg false discovery rate (FDR) procedure, and adjusted *P*-values <0.05 were considered significant for branch-site model analysis. We used the same orthologous genes and tree topology as for PSGs to identify rapidly evolving genes (REGs). The branch model in PAML was used, with the null model (model = 0) assuming that all branches have been evolving at the same rate and the alternative model (model = 2), allowing the foreground branch to evolve under a different rate. An LRT with df = 2 was used to discriminate between alternative models for each ortholog in the gene set. Genes with adjusted *P*-values <0.05 after multiple test corrections using the FDR program for the foreground (ancestral branch of waterfowl) in relation to the background branches were considered as evolving with a significantly faster rate in the foreground branch. The PSGs, REGs, and expanding and contracting gene families obtained above were all treated as separate gene sets for enrichment analysis with WebGestalt (Elizarraras et al. 2024).

### Exon codon tree with 1st and 2nd positions

Considering the composition heterogeneity of different codon positions, we further partitioned the orthologous gene sequence into 1st, 2nd, and 3rd codon positions. We partitioned the 1st, 2nd, and 3rd codon positions separately, such that the rate matrices were estimated based on different codon positions, but the branch lengths were estimated jointly (Emms and Kelly 2019).

### Whole-genome alignments

Whole-genome alignments (WGAs) are critical for comparative analyses, and we generated 25-way multiple genome alignments for all the 24 waterfowl species and chicken. WGAs of 24 waterfowl species with chicken (GCA_000002315.5) as reference were constructed by ProgressCactus (v.2.8.0) to produce a HAL alignment file (Armstrong et al. 2020). The HAL file alignment was converted to MAF-format alignments using the HAL tools package (V.2.1) (Hickey et al. 2013). Segments in the final merged MAF file that were lacking more than 13 species were discarded. Then, all segments were concatenated by means of a perl script. To reduce the computational requirement, genome sequences were soft-masked and contigs shorter than 100 kb were discarded. A total of 47.1 Mb autosomal syntenic sequence and 2.18 Mb ChrZ syntenic sequence are shared across all waterfowl genomes and the outgroup species. ML phylogenetic tree constructed using CDS dataset was used as guide tree in WGAs.

### Identification of conserved non-exonic elements CNEEs and 4-fold degenerate (4d) sites

First, to estimate the nonconserved model of 24 waterfowl genomes and the chicken genome, we employed phyloFit (V.1.4) in the PHAST package with 4d sites in the waterfowl alignments and the topology (Wishart et al. 2023). Then, we ran phastCons with the waterfowl nonconserved model to estimate the waterfowl conserved models. Also, with phastCons, we predicted the highly conserved elements and generated base wise conservation scores with the waterfowl conserved and nonconserved models. Exon regions were excluded from the highly conserved elements to generate the conserved non-exonic elements (CNEs). We identified 358,614 CNEs and filtered out CNEs smaller than 20 bp, resulting in a total length of 34 Mb CNEs data class. The 4d sites of 25-way WGAs were extracted by using hal4d and maftools (Armstrong et al. 2020). In total, 716,848 sites were catenated.

### Whole-genome ML tree

WGAs of 24 waterfowl genomes and the chicken genome were used to construct a phylogenetic tree rooted by the chicken. Syntenic blocks were concatenated using maftools, and a PHYLIP-formatted alignment file containing ∼178 Mb sequences for each genome was then generated. Exascale Maximum Likelihood (ExaML) (v.3.0.17) was used to handle the large data set (Kozlov et al. 2015). Twenty guidance trees (randomized stepwise addition order parsimony trees) were generated using RAxML (v.8.2.9) with the “GTRCAT” model and a random seed value (Stamatakis 2014). Each of the 20 guidance trees was inputted for ML analysis. A final ML tree was generated by selecting the highest likelihood value from the 20 ML trees. To perform bootstrap analysis, 100 bootstrap alignments were generated using RAxML. Each of the 100 bootstrapped alignments was used to generate a guide tree with “GTRCAT” model by RAxML and converted into a binary file using “parse-examl” command. Each pair of the guide tree and the binary file generated from 100 bootstrapped alignments were inputted for ML analysis with ExaML and generated trees with branch lengths. These trees with branch lengths were used to calculate the bootstrap values based on the selected final ML tree. Finally, we obtained an ML tree with BS on each node.

### Phylogenetic tree inferred by NJst and Raxml for waterfowl DNA data set

We applied NJst, a fast, accurate “two-step” species tree method based on the NJ algorithm and derived from the multispecies coalescent model, which can infer a species tree from rooted gene trees. NJ phylogenetic trees for all seven DNA data classes were inferred using NJst (Liu and Yu, 2011). ML trees of six DNA data classes except WGAs were constructed using RAxML (v8.2.9) (176) with 100 bootstrap replicates under the GTRGAMMA model. The resulting tree with the highest likelihood score was selected as the best tree.

### Coalescent-based genome tree inference

Standard concatenation approaches may not completely model the discordance among gene trees beyond differences in sequence evolution rates (Wu, 2015). Previous studies have also shown that ILS could lead to incorrect topology, possibly due to estimation bias in concatenated analyses where the mixture of gene trees represents a model violation. These possible limitations can theoretically be overcome with multispecies coalescent methods using, e.g. ASTRAL. Therefore, after constructing ML trees using RAxML for each CDS nucleotide sequence, all trees were parsed by ASTRAL-III (v5.5.4) to obtain the coalescent-based consensus species tree (Zhang et al. 2018). Standard concatenation methods may not fully simulate inconsistencies between gene trees other than differences in sequence evolution rates (Wu, 2015). Previous studies have also shown that ILS can lead to topological errors, which may be caused by estimation bias in concatenation analysis, because in this case, the mixing of gene trees represents model violations. In theory, these possible limitations can be overcome by using multi-species merging methods such as ASTRAL. Therefore, after using RAxML to build ML trees for each CDS nucleotide sequence, all trees were parsed using ASTRAL-III (v5.5.4) to obtain a consensus species tree based on merging (Zhang et al. 2018; Chen et al. 2019). In addition, we also split the WGAs into non-overlapping windows of 100 kb, and RAxML built ML trees for the nucleotide sequences of each window and then used ASTRAL-III to obtain a consensus species tree based on merging. Then, we randomly selected five major taxa of waterfowl as representatives (*Anatinae*, *Anserinae*, *Dendrocygna*, *Anseranatidae* and *Anhimidae*) and used DensiTree to draw density trees (Bouckaert 2010).

### Divergence time calibration

We used the 4d sites DNA data class (∼0.71 Mb sequences) in MCMCTREE to estimate the divergence times within waterfowls. The divergence times of Galliformes ∼ Anseriformes and Anatinae ∼ Anserinae as conservative time constraints were used to calibrate the external tree in MCMCTREE (Yang 2007). The divergence time of the common ancestor of Galliformes and Anseriformes has yielded similar results in multiple studies, so the divergence time of the two orders (∼72.5 to 85.4 Mya) was used as the calibration point. In this range, consensus is found with van Tuinen and Hadly (2004) for the higher end of this range and with Claramunt and Cracraft (2015) for the lower range. As the clade with the largest number of species in this study, the divergence time of Anatinae and Anserinae (∼22.4 to 36.0 Mya) was used as another calibration point. Maximum age for crown Anatidae was derived from stem Romainvillidae in the Late Eocene, and minimum age from oldest crown Late Oligocene anatid fossils (Mayr 2009). Minimum-maximum ages were input as prior in divergence time analysis. Posterior distributions of divergence times were estimated by Markov chain Monte Carlo sampling, with samples drawn every 2,000 steps over a total of 108 steps after a discarded burn-in of 107 steps. To check for convergence of the stationary distribution, each analysis (including the “baseml” step) was run in duplicate, and the results were compared between runs. The nucleotide substitution rates (in units of substitutions per site per million years) in waterfowl genomes were estimated by comparisons of the fourfold degenerate (4d) sites in coding regions and divergence times between waterfowl species.

### Phylogenetic discordance analyses

Many factors could give rise to incongruent tree topologies among different classes of DNA datasets. Here, we used a recently published method to evaluate the relative contributions of ILS, tree estimation error and gene introgression to phylogenetic heterogeneity in waterfowl (Cai et al., 2020). The method was applied to two DNA datasets in waterfowl: CDS dataset and 100 kb non-overlapping sequence windows from WGAs. First, we quantified tree topological variation across the species tree by computing gCF using IQ-TREE (v.2.2.2). For every branch of a reference tree, gCF is defined as the percentage of “decisive” gene trees containing that branch (Minh et al. 2020). Second, we quantified tree inference error via alignment simulation, tree estimation, and bipartition summarization. The 200 alignments simulation under the GTR model was implemented in SeqGen (v.1.3.5) with sequence length of 1,500 bp single copy gene dataset and 100 kb for non-overlapping window sequences. The bipartition summarization was implemented in RAxML. Third, we quantified the degree of ILS in each node of the species tree using a “Theta” value for each node. Based on the concatenation supermatrix, we estimated the branch length for each node in mutation unit using RAxML, and in coalescent unit inferred from ASTRAL-III. “Theta” for each node was determined by dividing the branch lengths from RAxML by that from ASTRAL-III. Fourth, we quantified the degree of potential gene introgression for each node using a reticulation index (https://github.com/lmcai/Coalescent_simulation_and_gene_flow_detection). Finally, we formatted the data above into a matrix, and used R package (relaimpo) to carry out the regression analysis. Several methods, including lmg, last, first, pratt, are capable of dealing with correlated regressors. lmg (Lindeman-Merenda-Gold) is considered the most robust method among these methods, and therefore described in the results section. Comparative results of other methods are displayed in S14 and S15.

### Topological conflict, ILS, and gene introgression

CoalHMM aims to predict the coalescent process back in time by fitting a hidden Markov model on estimates of ancestral population genetics parameters (Dutheil et al. 2009). The hidden states of CoalHMM are four different topologies (Fig. 3e), namely the species tree topology (V0), the deep coalescent topology following the species tree (V1), or one of two alternative topologies incongruent with the species tree (V2 and V3). The observed states are columns along a 4-way genome alignment with three species and an outgroup. We can calculate the probability of a site belonging to each of the four hidden states of the HMM using posterior decoding. This way, we can obtain a base-pair-level resolution on the distribution of ILS along the genome. In this study, we investigated the reasons for four gene tree incongruences using the autoCoalHMM pipeline which divides the WGAs into 100 kb windows and performs topological analysis on three species and one outgroup (Rivas-González et al. 2023). The horizon plots were then built using the R package ggHoriPlot (https://github.com/rivasiker/ggHoriPlot).

Each species topology represents a different pair of two most closely related species. If ILS is the main source of incongruent topologies for a given species quartet, we expect estimates of genetic divergence between these two species to be smaller for the species topology (V0) than for the two alternative topologies (V1: species1/species2, V2: species1/species3, or V3: species2/species3). This is because genomic segments attributed to alternative topologies can only coalesce in the common ancestor of all three species. However, if ancestral introgression is the main source of incongruencies, we expect the opposite pattern, with the alternative topologies V2 or V3 showing the smaller divergence between the closest species pairs. Additionally, in this case, we expect asymmetry between the divergence level of V2 versus V3 segments, an asymmetry of the total proportion of the genome attributed to V2 or V3, and the distribution of V2 vs V3 segments lengths, whereas these two alternative topologies are expected to be equally frequent under an ILS model (Rivas-González et al. 2023). To assess whether the level of incongruences we report in this study is consistent with ILS in each branch of the phylogeny, we compared the level of divergence between sister species for segments attributed to V0, V1, V2, or V3 state, individually in each branch.

It is challenging to detect gene introgression on such a protracted time scale as that concerning waterfowl evolution. As the divergence time of all waterfowl is ancient, the commonly used methods based on linkage disequilibrium are not suitable. Here, we employed ABBA-BABA based analysis, which is relatively simple, direct and robust. We applied the classic ABBA-BABA test (*D*-statistics) using Dsuite (Malinsky et al. 2021). Vcf files of waterfowl species were converted from the WGAs MAF files using mafFilter. To examine possible gene introgression among three gene tree incongruences of waterfowl, we used black swan (*Cygnus atratus*) and chicken (*Galliformes*) as the outgroup (O), respectively. Significant *Z*-scores (*Z* ≥ 3, after Bonferroni correction for multiple testing) indicate evidence of test population P3 containing an admixture of reference populations of P1.

In addition to the methods of Cai et al. (2020), autoCoalHMM and *D*-statistics, we used phytop (v.0.3) to assess the proportion of gene tree topological inconsistencies that can be explained by ILS and gene introgression (Shang et al. 2025). It defines ILS and IH indices to quantify the degree of ILS and gene introgression, which reflect gene tree heterogeneity on a single node basis. These four methods, although differing in underlying algorithm, provide a complementary perspective on the relative contribution of ILS and gene introgression to gene tree heterogeneity.

## Supplementary Material

msag018_Supplementary_Data

## Data Availability

The data classes presented in this study can be found in online repositories. The genome assembly was deposited at NCBI with BioProject accession number PRJNA1289077.
