# Supplementary material for: Phylogeny of waterfowl (Anseriformes) constructed using genome sequences provides insights into topological incongruences
Source: Mol Biol Evol. 2026 Jan 21;43(2):msag018. doi: 10.1093/molbev/msag018 (PMC12902361; doi:10.1093/molbev/msag018)
Supplement: msag018_Supplementary_Data [file msag018_supplementary_data.pdf]

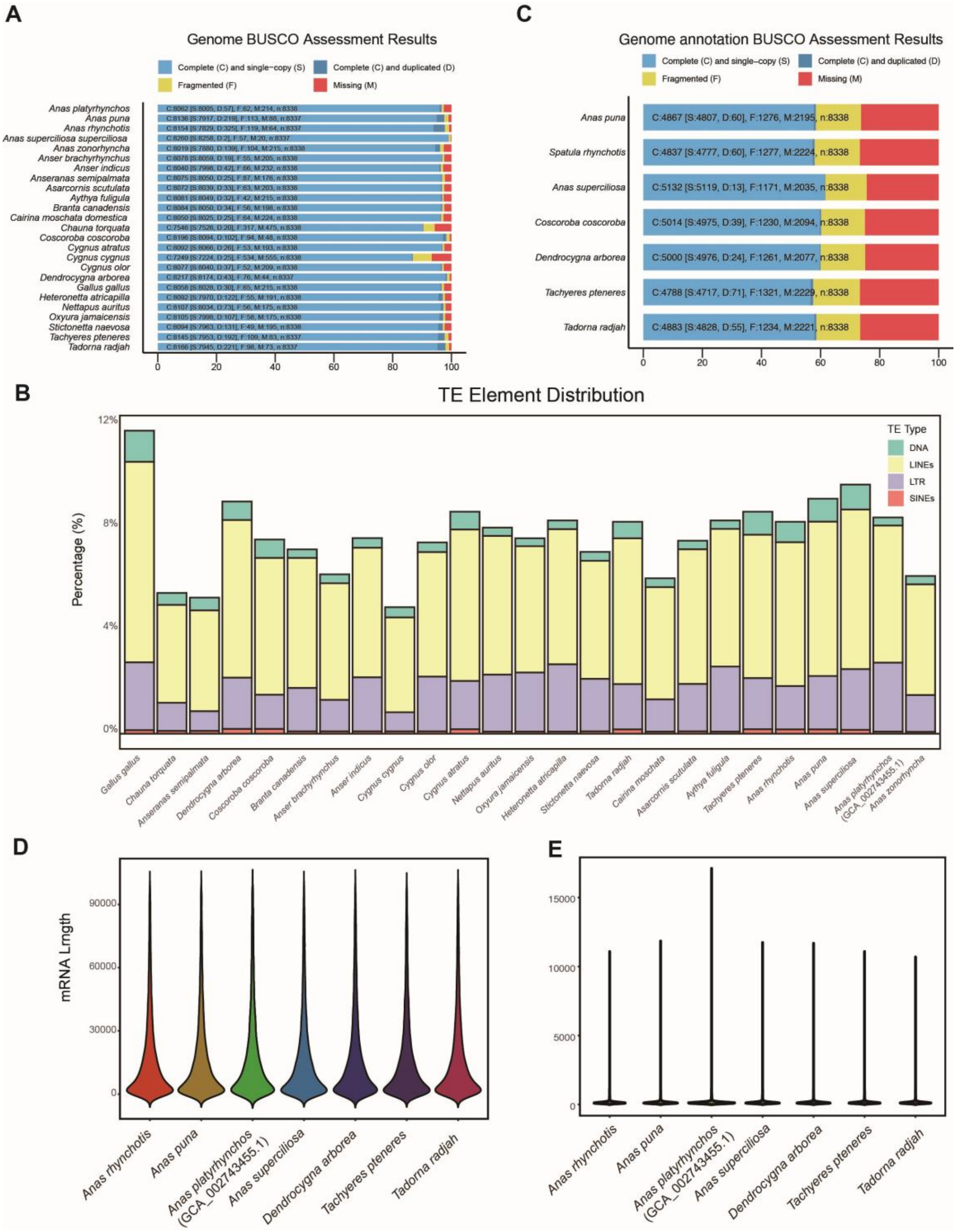

Figure S1. Genome quality and completeness for phylogenetic analysis and Transposable Element distribution (TEs) in the genome. (A) Completeness of BUSCO assessment for all species used in this study, including 24 waterfowl and 1 outgroup. (B) The contents of different TE types of 24 waterfowl and chicken. (C) Completeness of BUSCO assessment for annotation of seven newly assembled waterfowl species genomes. (D) and (E) are the mRNA and CDS length distributions of seven newly assembled waterfowl genomes , respectively. The mallard duck assembly CAU_duck1.0 (GCA_002743455.1) is compared with the newly assembled genome.

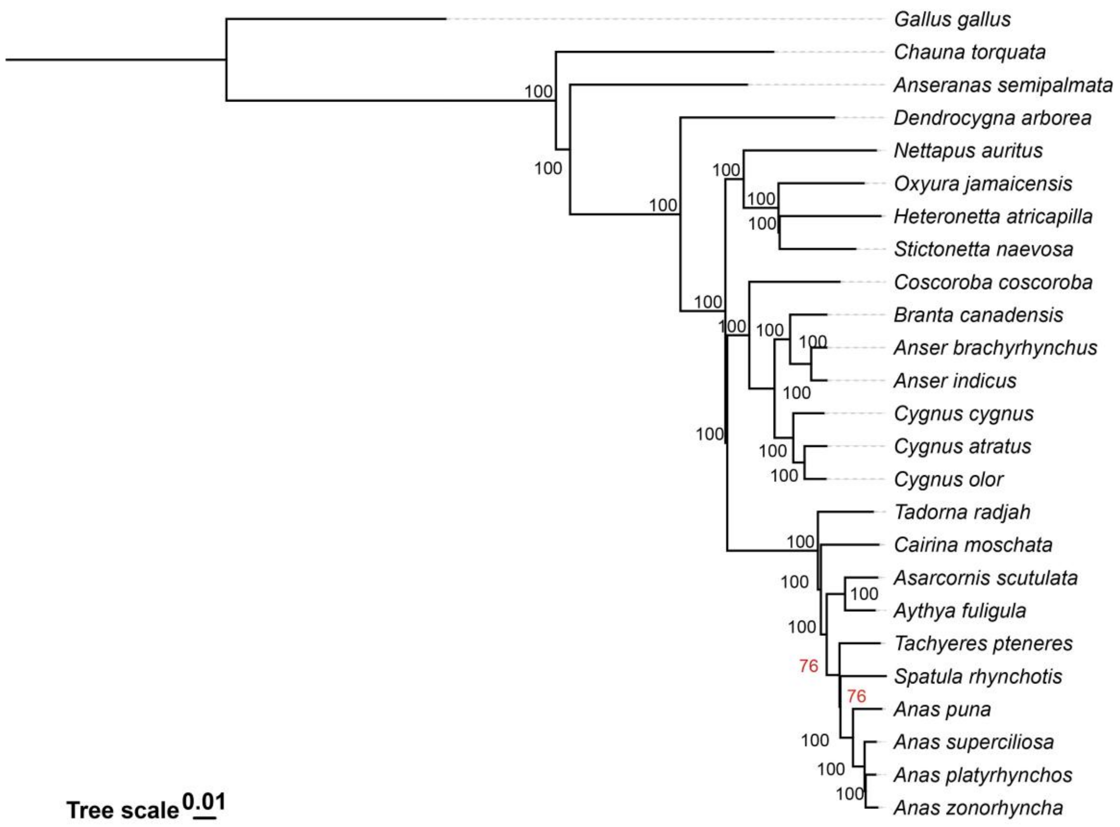

Figure S2. Waterfowl Whole-genome alignments (WGAs) phylogenetic tree constructed using NJ method.

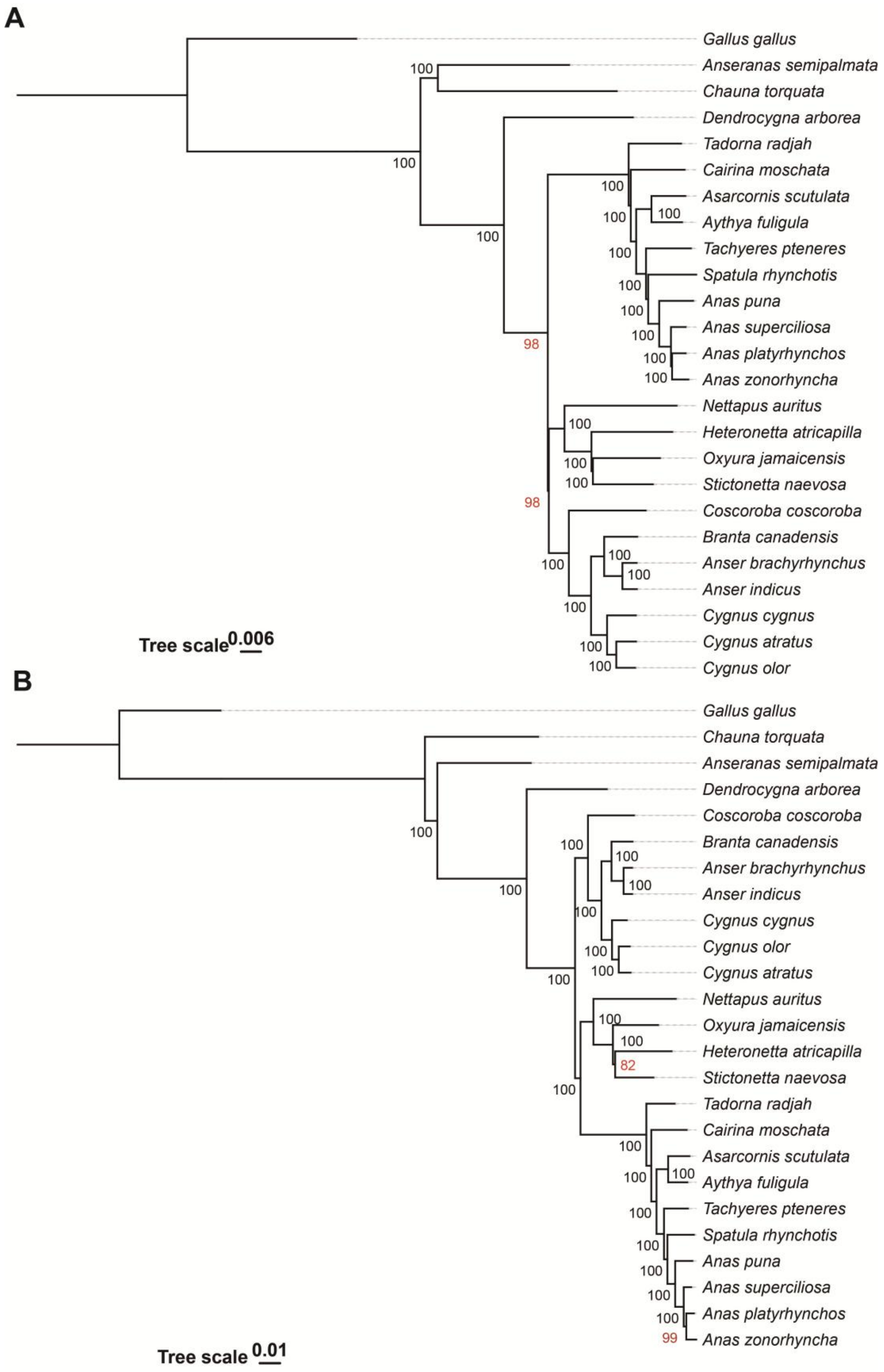

Figure S3. Waterfowl Four-fold degenerate (4d) sites phylogenetic tree using different methods. (A) Waterfowl Four-fold degenerate (4d) sites tree using NJ method; (B) Waterfowl Four-fold degenerate (4d) sites tree using ML method.

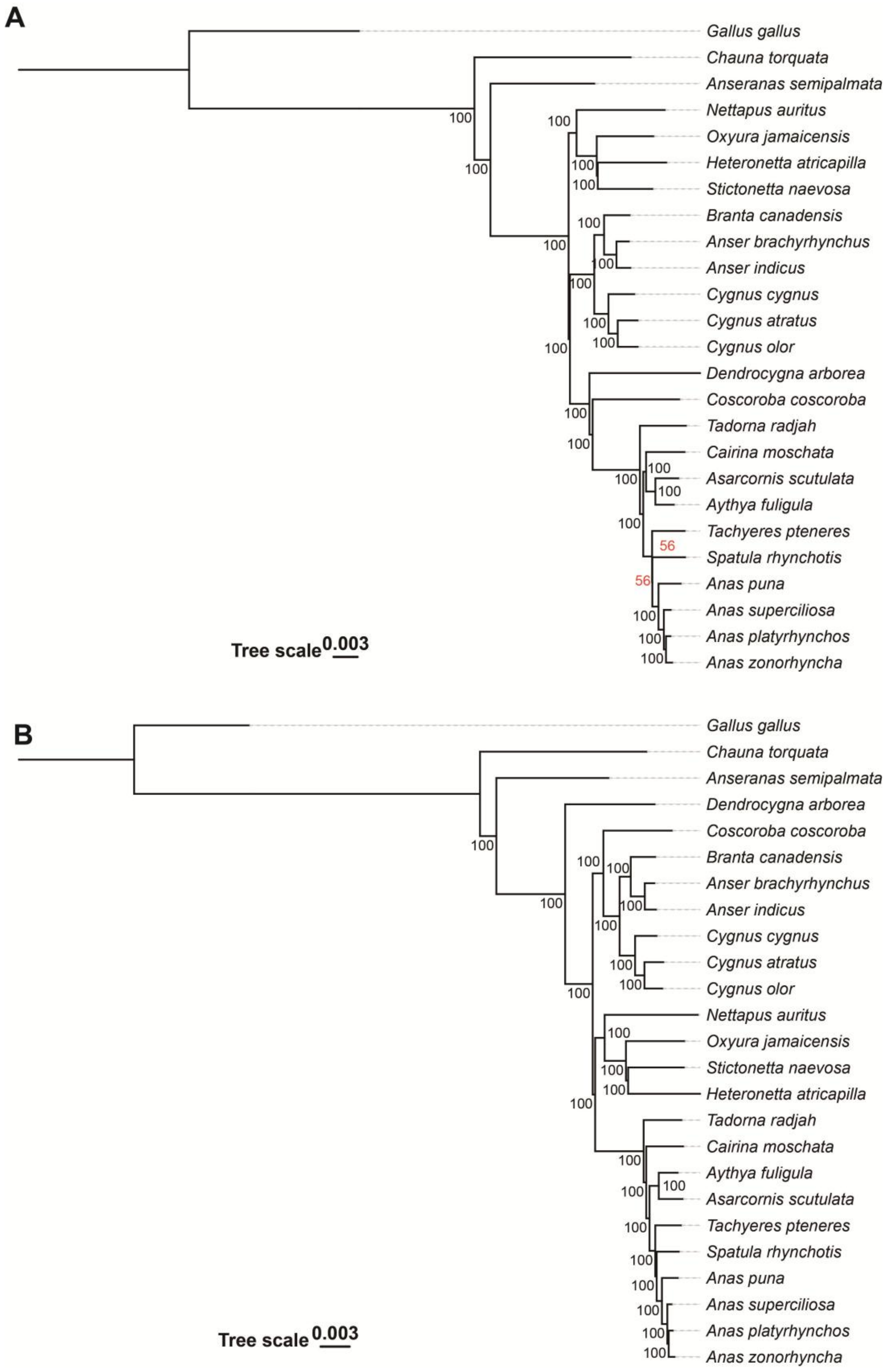

Figure S4. Waterfowl Conserved non-exonic elements (CNE) phylogenetic tree using different methods. (A) Waterfowl CNE tree using NJ method; (B) Waterfowl CNE tree using ML method.

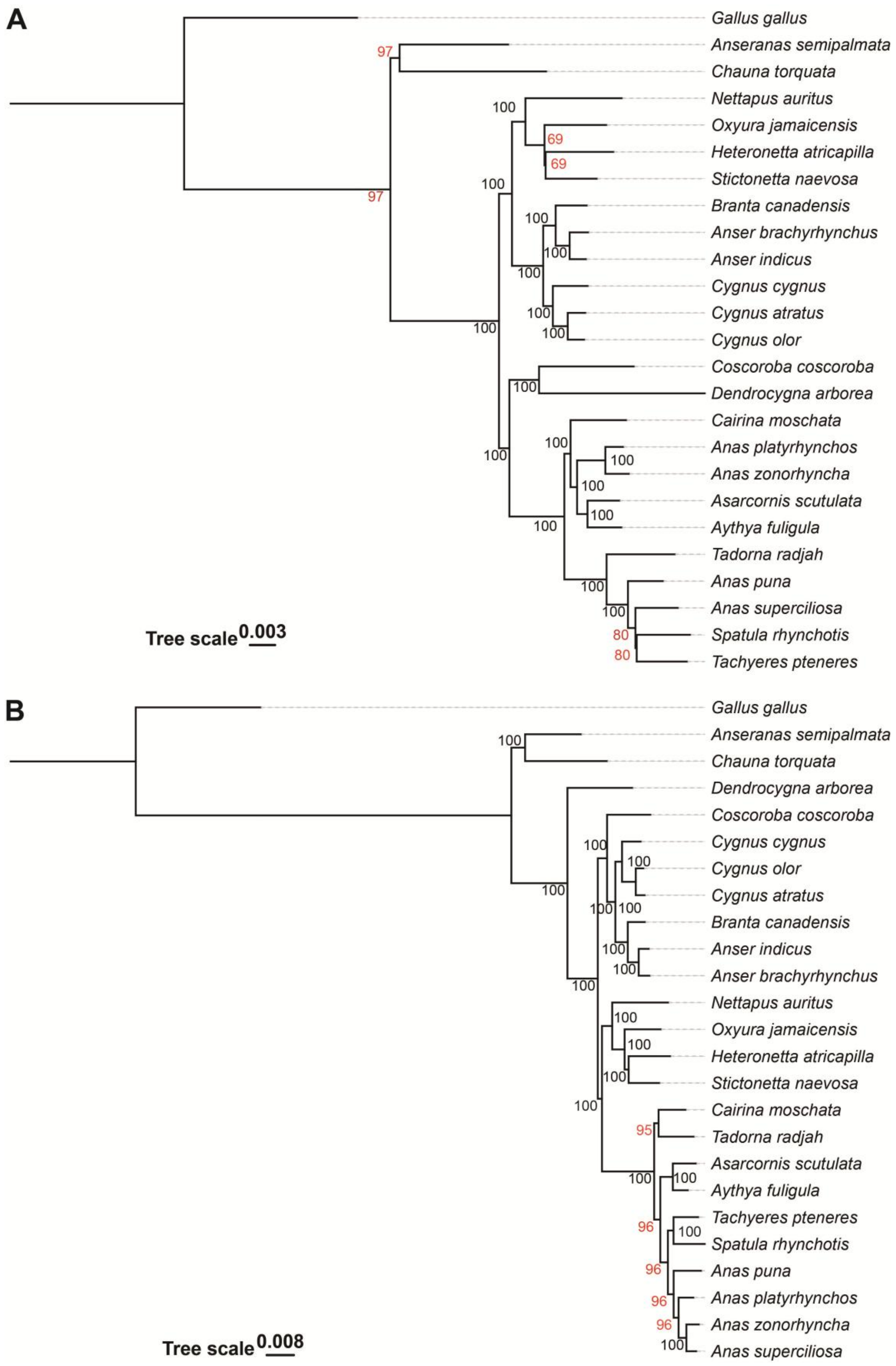

Figure S5. Waterfowl Codon1&2 phylogenetic tree using different methods. (A) Waterfowl Codon1&2 sites tree using NJ method; (B) Waterfowl Codon1&2 tree using ML method.

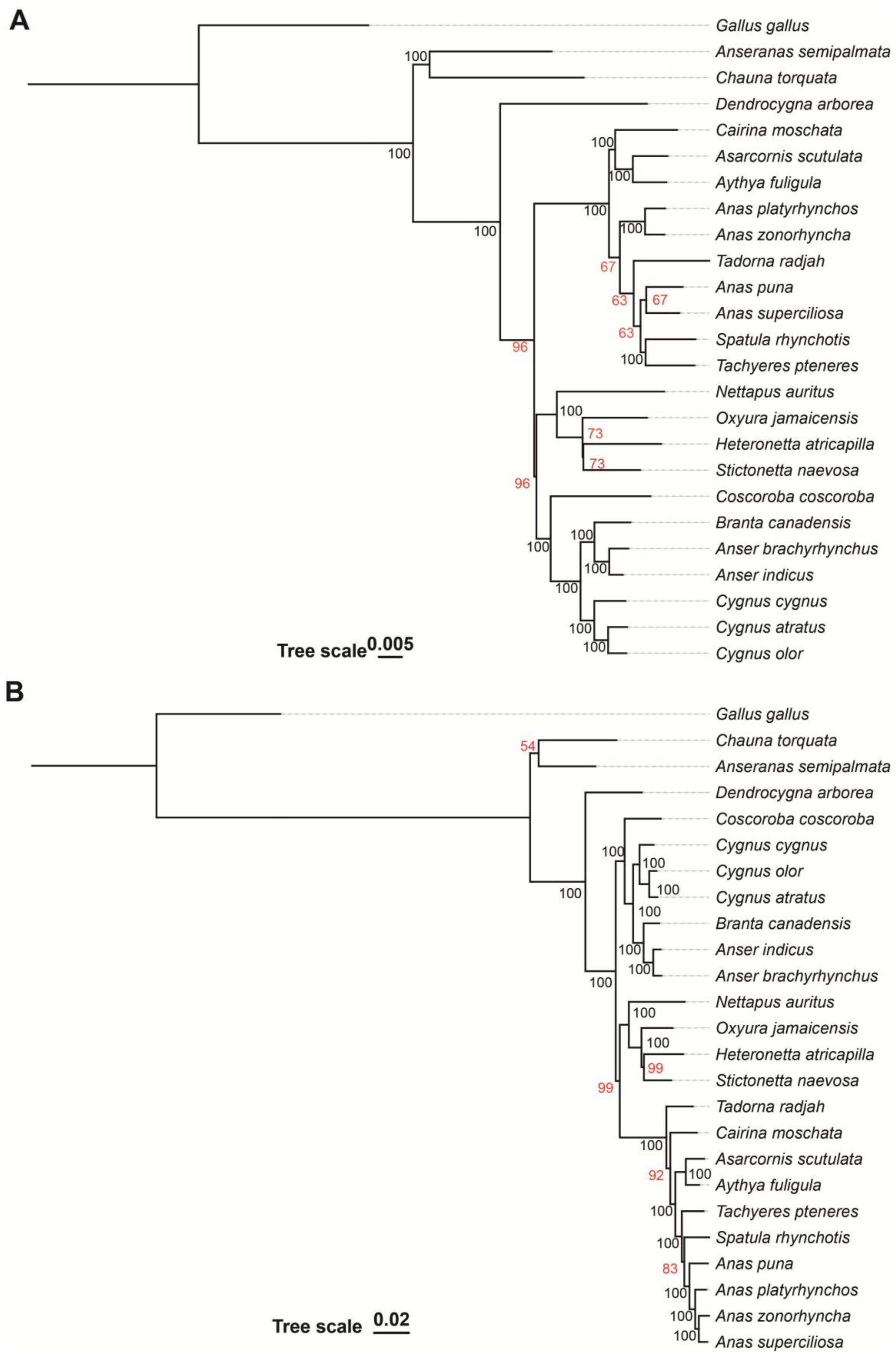

Figure S6. Waterfowl Codon3 phylogenetic tree using different methods. (A) Waterfowl Codon3 tree using NJ method; (B) Waterfowl Codon3 tree using ML method.

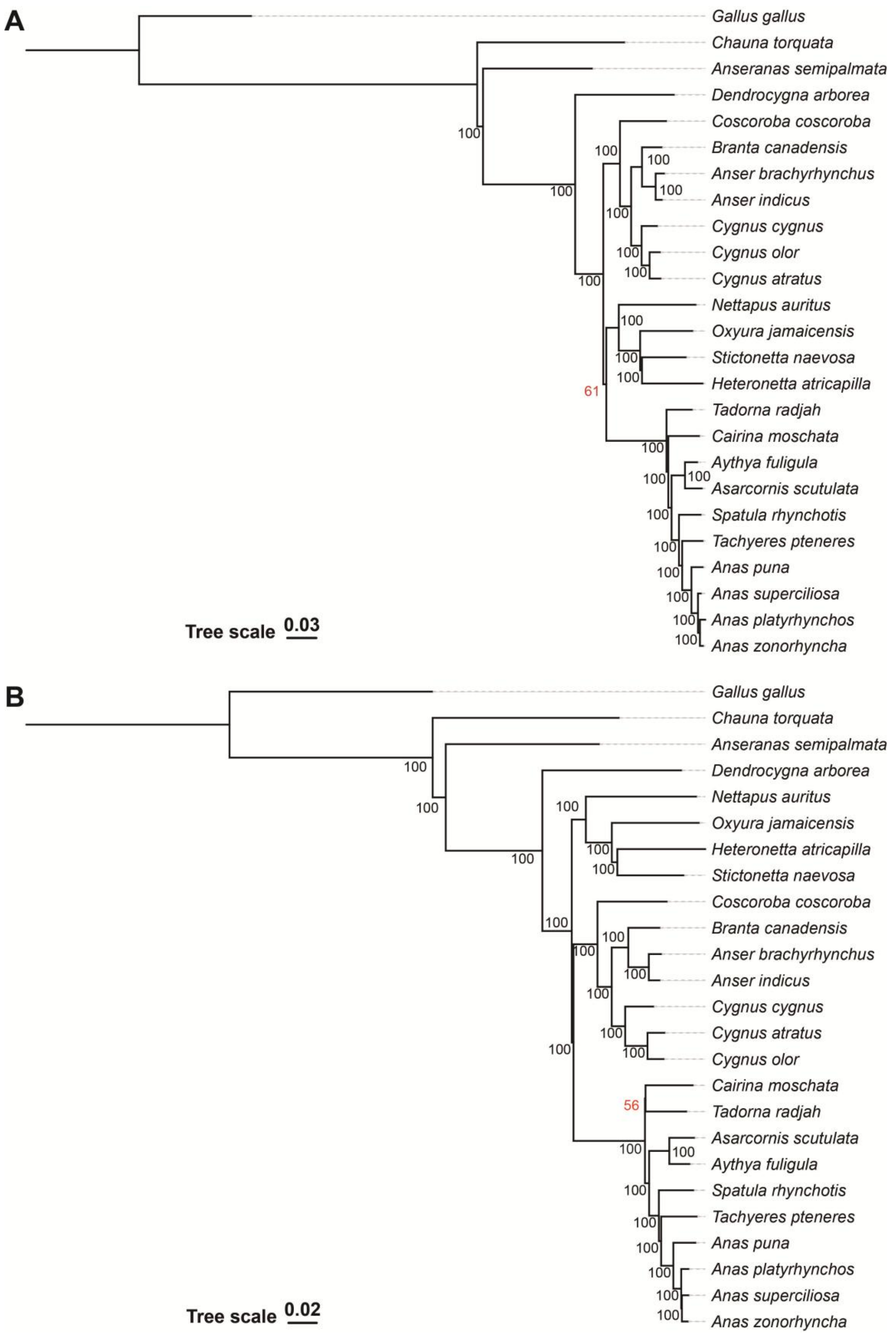

Figure S7. Waterfowl ChrZ phylogenetic tree using different methods. (A) Waterfowl ChrZ tree using NJ method; (B) Waterfowl ChrZ tree using ML method.

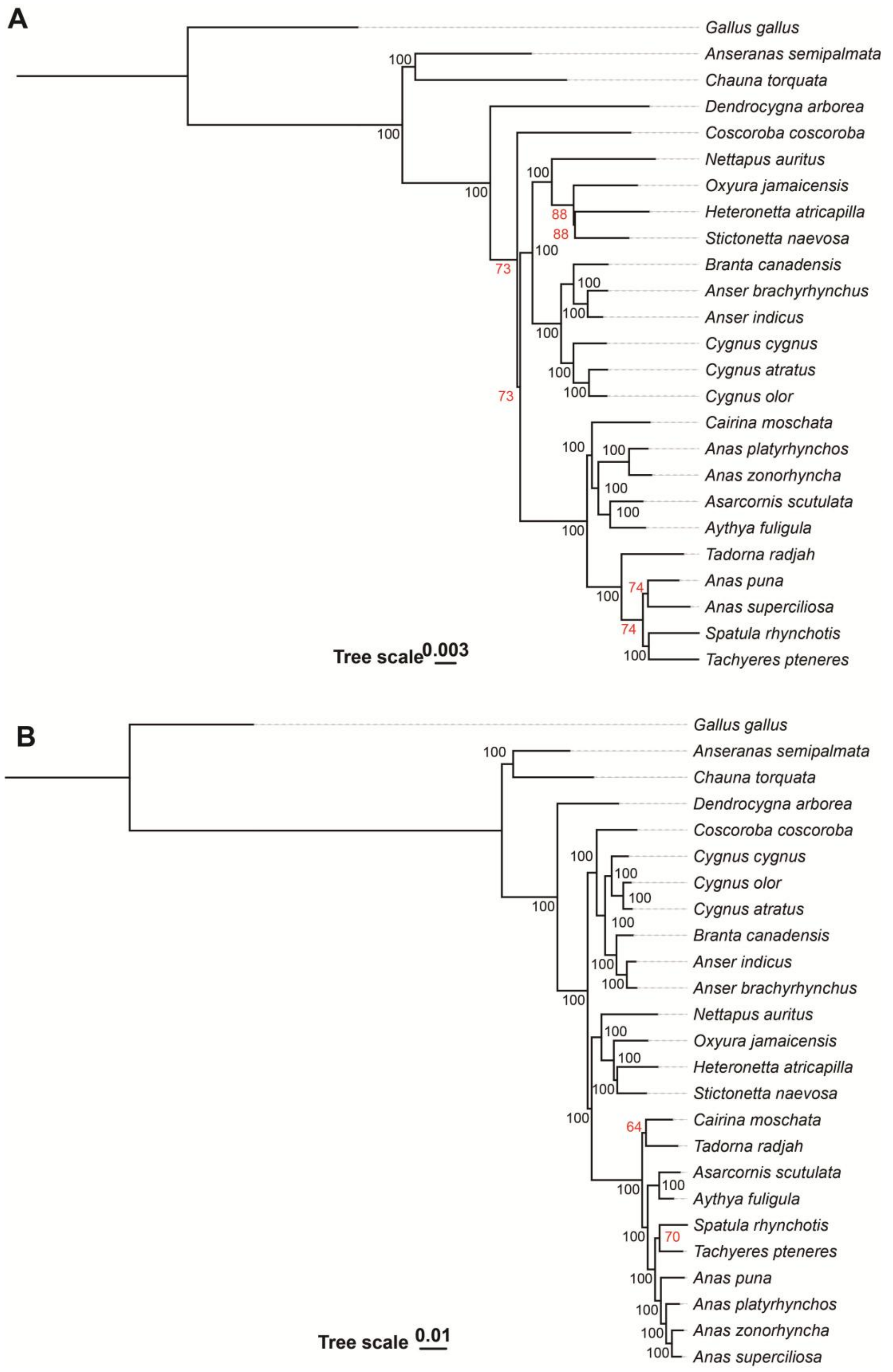

Figure S8. Waterfowl homologous single copy genes coding sequences (CDS) phylogenetic tree using different methods. (A) Waterfowl CDS sites tree using NJ method; (B) Waterfowl CDS tree using ML method.

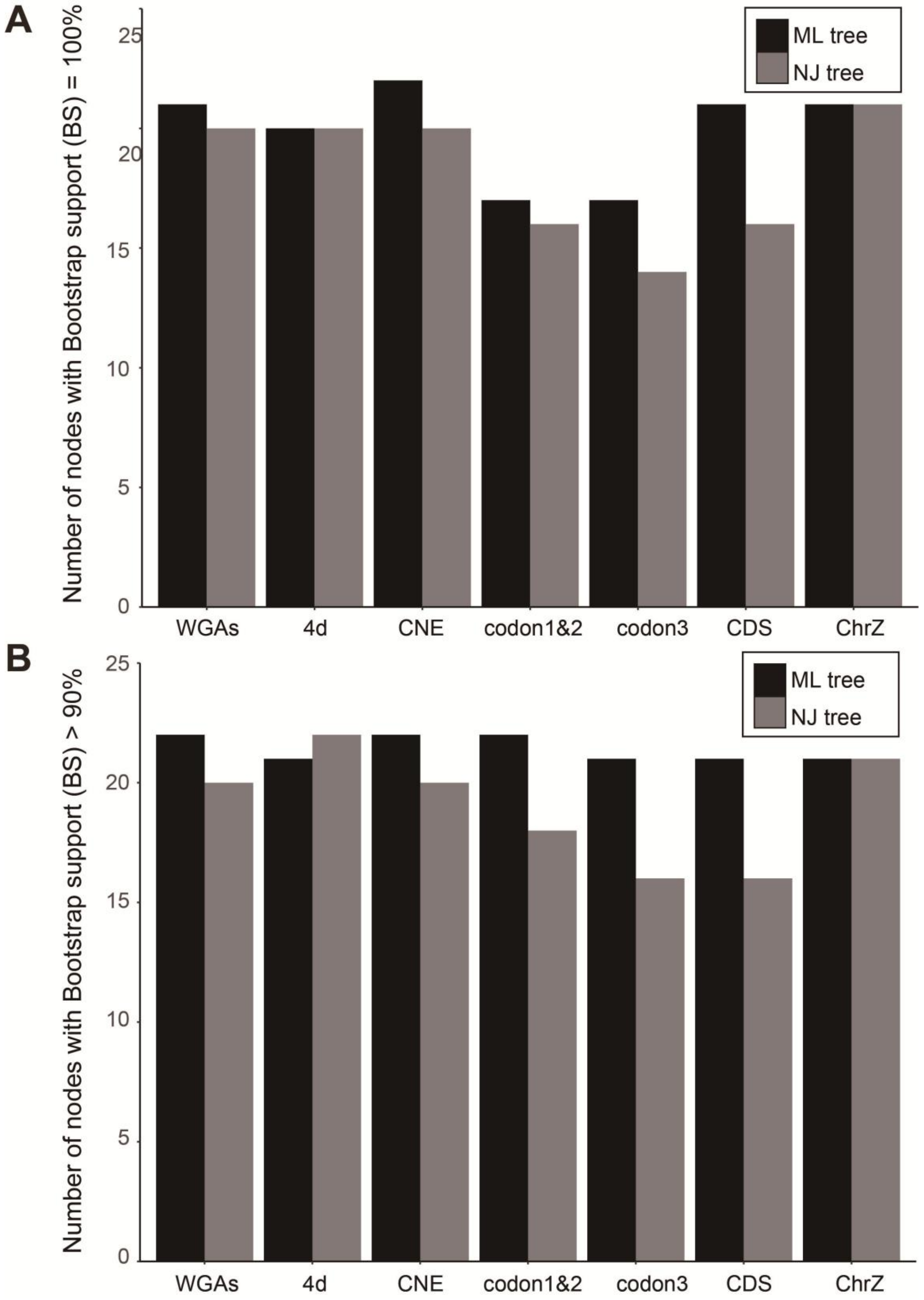

Figure S9. Comparison of the number of significant consensus nodes (BS=100% (A) and BS>90% (B)) among 22 nodes for trees estimated using NJ and ML algorithms across all seven pairwise combinations of DNA classes.

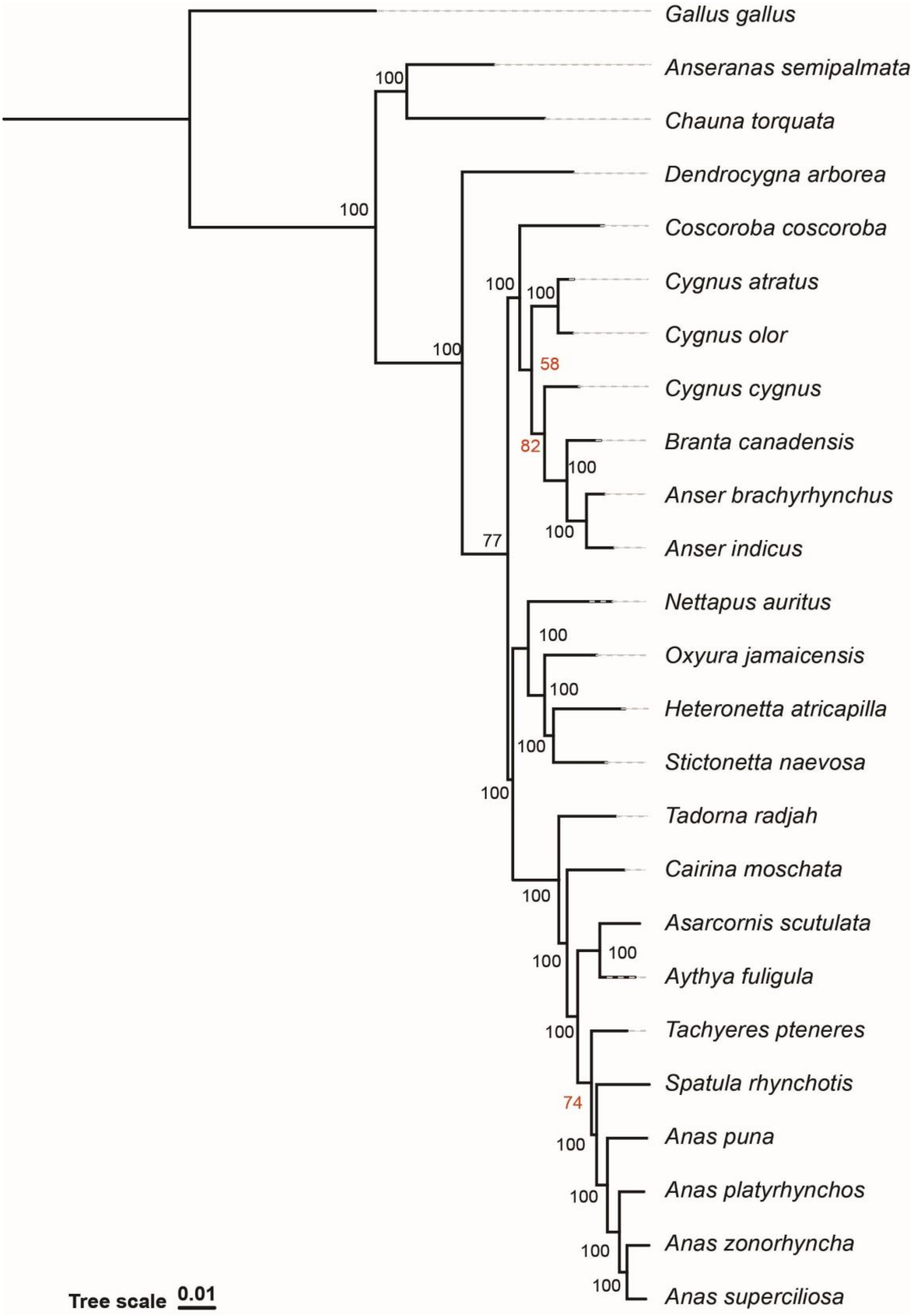

Figure S10. Waterfowl homologous single copy genes coding (CDS) protein sequence ML phylogenetic tree using IQ-TREE with Q.mammal+F+I+I+R6 substitution model.

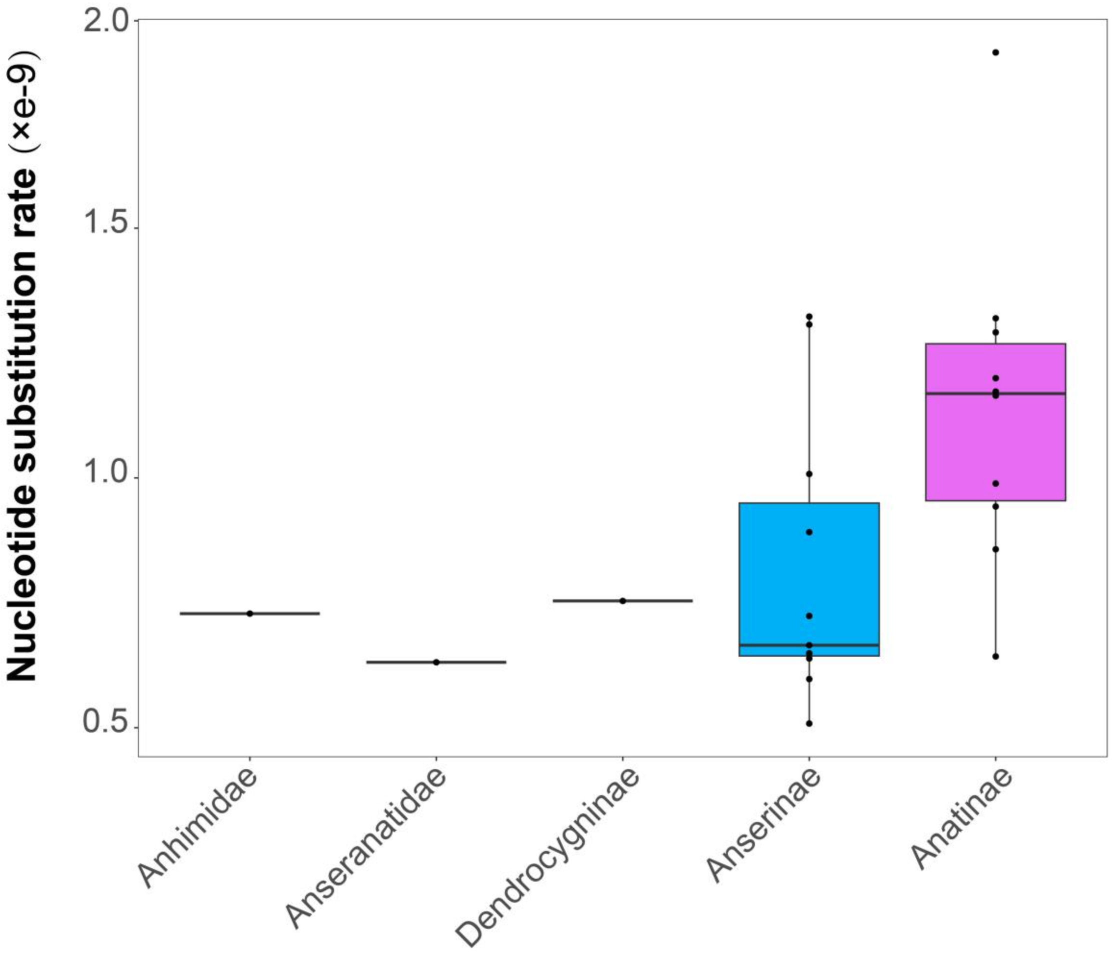

Fig S11. Substitution rates across five major taxa of waterfowl.

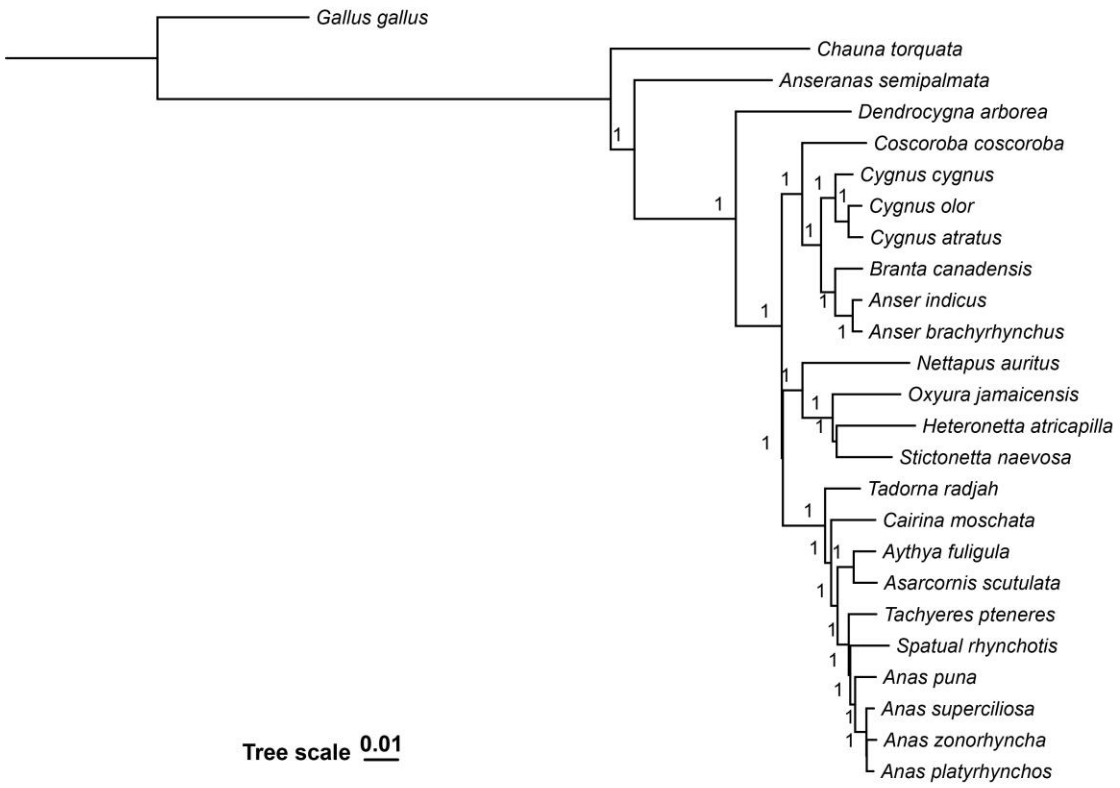

Figure S12. Waterfowl 100 kb non-overlapping window WGAs coalescent tree using astral method.

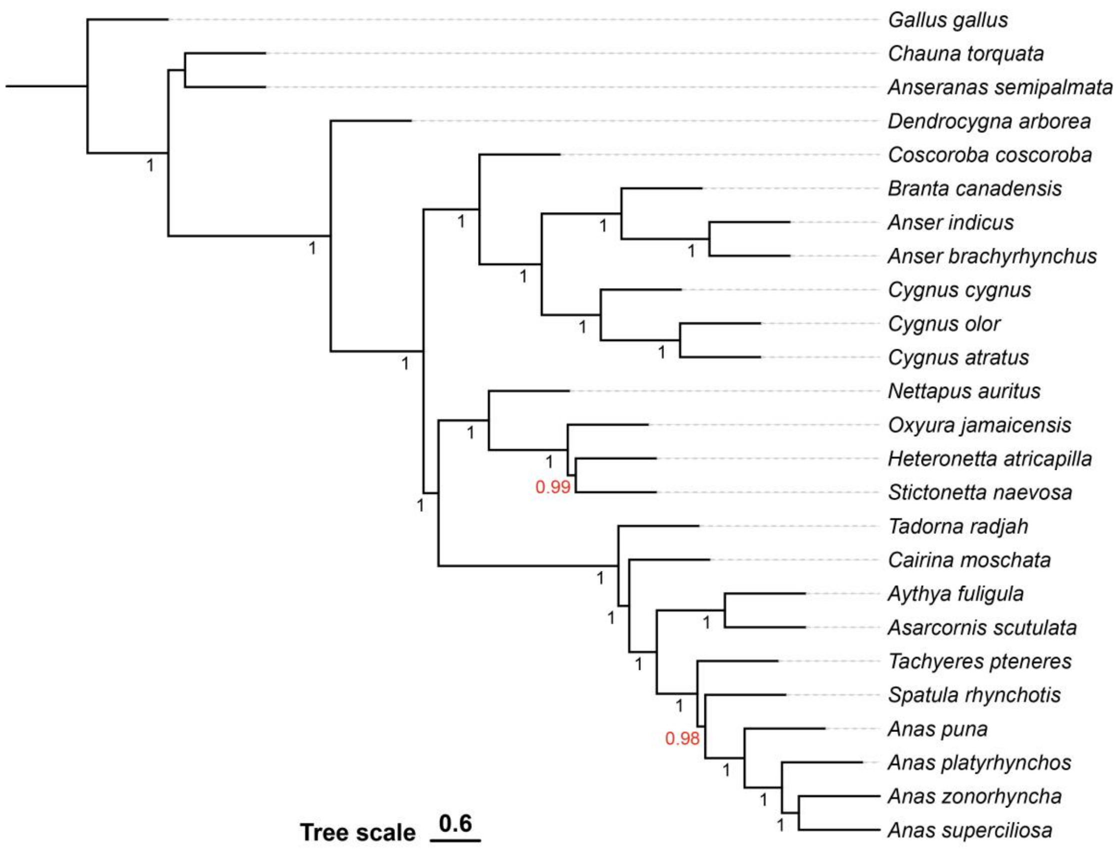

Figure S13. Waterfowl homologous single copy genes coding sequences (CDS) coalescent tree using astral method.

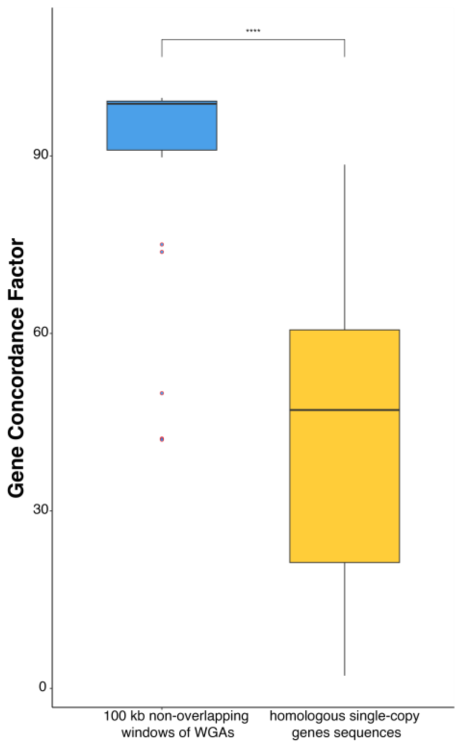

Figure S14. Box plots of gCF of all nodes based on homologous single-copy gene coding sequences (CDS) and 100 kb non-overlapping window WGAs by using IQ-TREE.

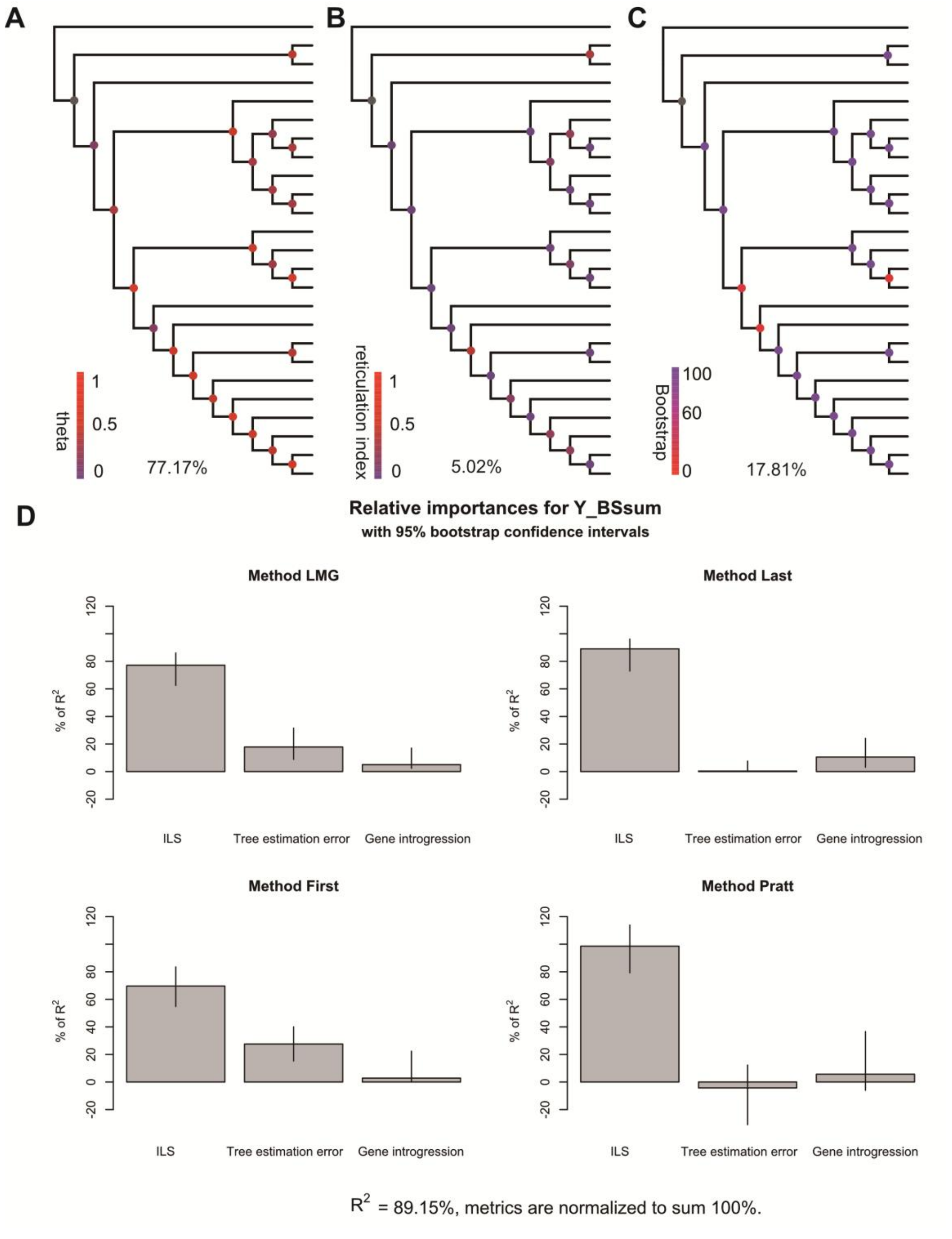

Figure S15. Analysis of waterfowl phylogenetic tree heterogeneity in CDS datasets . (A) ILS. Nodes are colored by inferred population mutation parameter theta. (B) Gene introgression. Nodes are colored by Reticulation Index. (C) Gene tree estimation error. Nodes are colored by bootstrap (BP) values, which represent percentage of recovered nodes from simulation. (D) Relative importance of incomplete lineage sorting (ILS), tree estimation error, and gene introgression in generating homologous single copy genes tree variation with Y_BSsum showing the gCF (Gene Concordance Factor) using IQ-TREE. The percentages are estimated based on four regression methods (LMG, Last, First, and Pratt) implemented in the R package relaimpo. 95% confidence intervals are represented by bars.

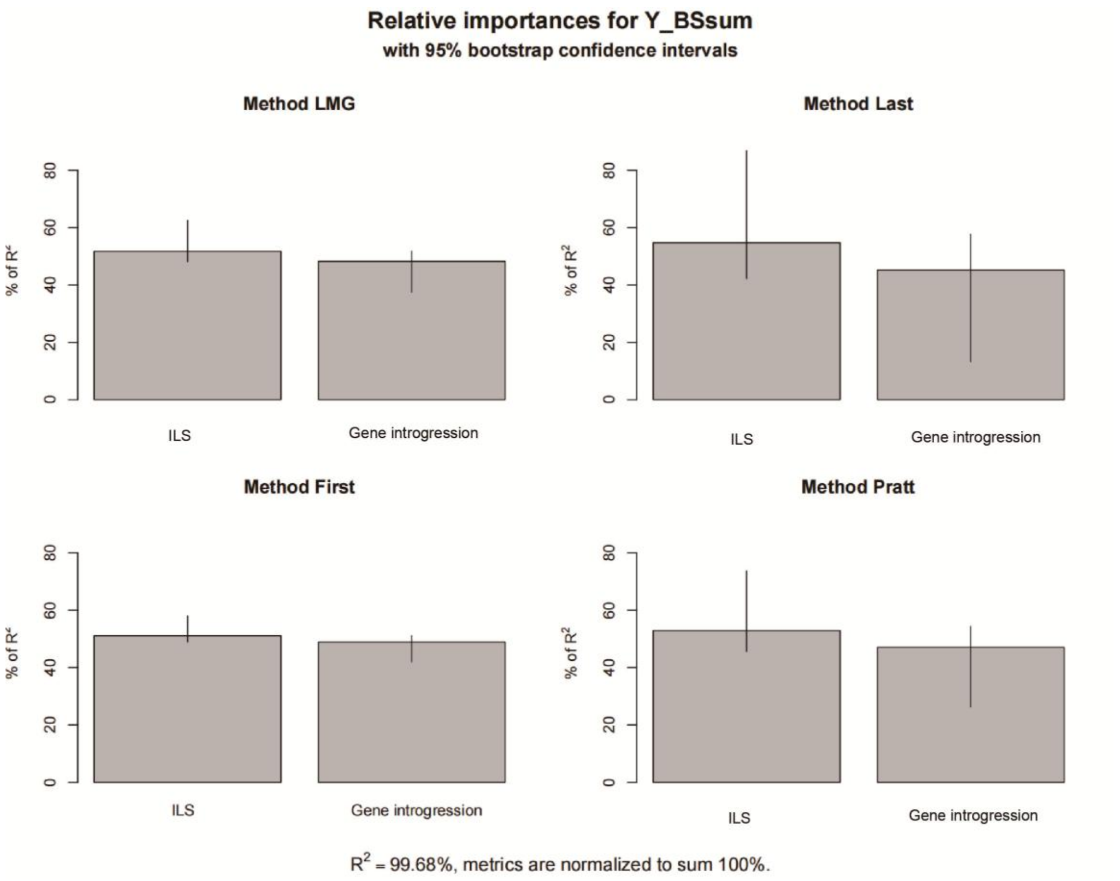

Figure S16. Analysis of waterfowl phylogenetic tree heterogeneity in 100 kb non-overlapping window WGAs. The percentages are estimated based on four regression methods (LMG, Last, First, and Pratt) implemented in the R package relaimpo. 95% confidence intervals are represented by bars. Y_BSsum shows the gCF (Gene Concordance Factor) using IQ-TREE.

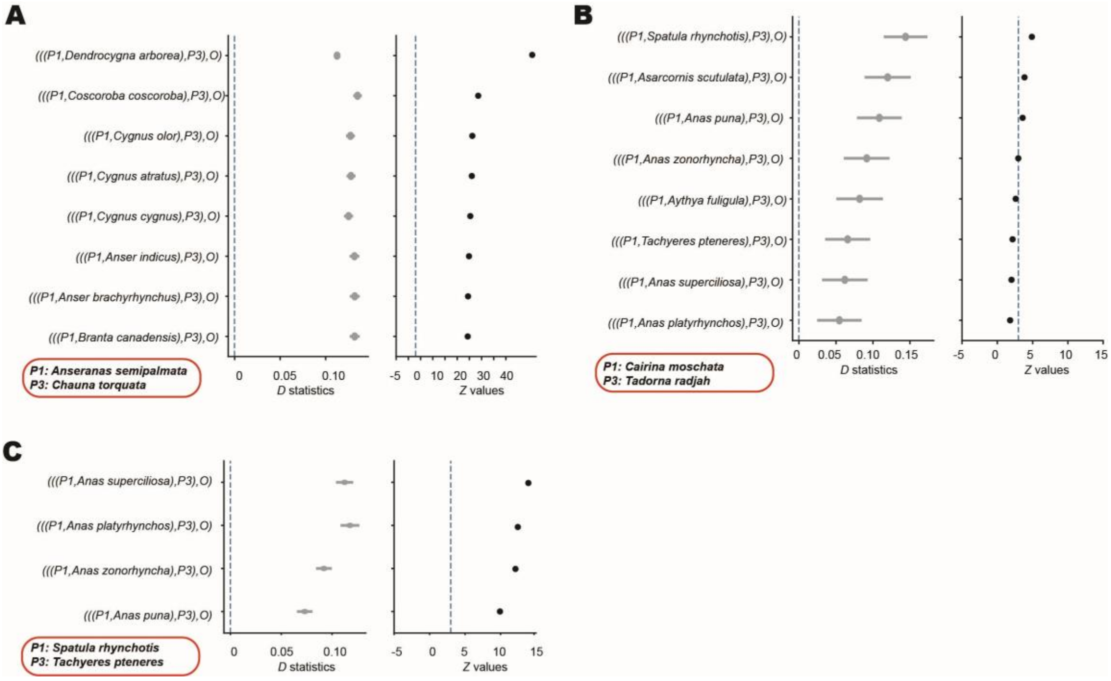

Figure S17. Analysis of gene introgression between waterfowl species using D-statistic. (A) Introgression analysis for the phylogenetic branching of *Anseranas semipalmata* and *Chauna torquata* with *Gallus gallus* as outgroup. (B) Introgression analysis for the phylogenetic branching of *Tadorna radjah* and *Cairina moschata* with *Cygnus atratus* as outgroup. (C) Introgression analysis for the phylogenetic branching of *Tachyeres pteneres* and *Spatula rhynchotis* with *Cygnus atratus* as outgroup.

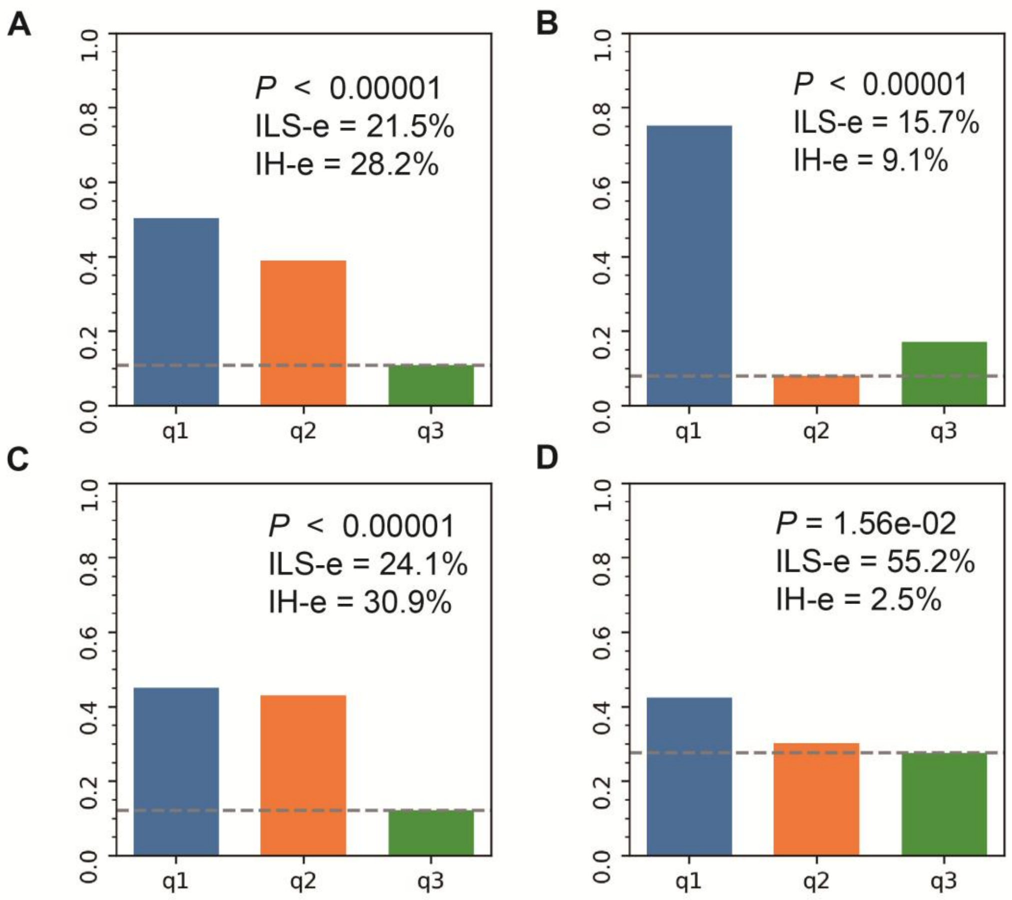

Figure S18. Analysis of ILS and gene introgression between waterfowl species using phytop. *P* is the *p*-value of $\chi 2$ test to check whether the number of topologies q2 and q3 are equal, and ILS-e and IH-e represent the proportion of gene tree topological incongruence that can be explained by ILS and gene introgression. Numbers below the branches represent the local posterior probabilities calculated in ASTRAL. (A) phytop analysis when the original combination of species is (Outgroup, (*Chauna torquata*, (*Dendrocygna arborea*, *Anseranas semipalmata*)). (B) phytop analysis when the original combination of species is (Outgroup, (*Anas platyrhynchos*, (*Tadorna radjah*, *Cairina moschata*))). (C) phytop analysis when the original combination of species is (Outgroup, (*Anas platyrhynchos*, (*Tachyeres pteneres*, *Spatula rhynchotis*))). (D) phytop analysis when the original combination of species is (Outgroup, (*Anas superciliosa*, (*Anas platyrhynchos*, *Anas zonorhyncha*)) .

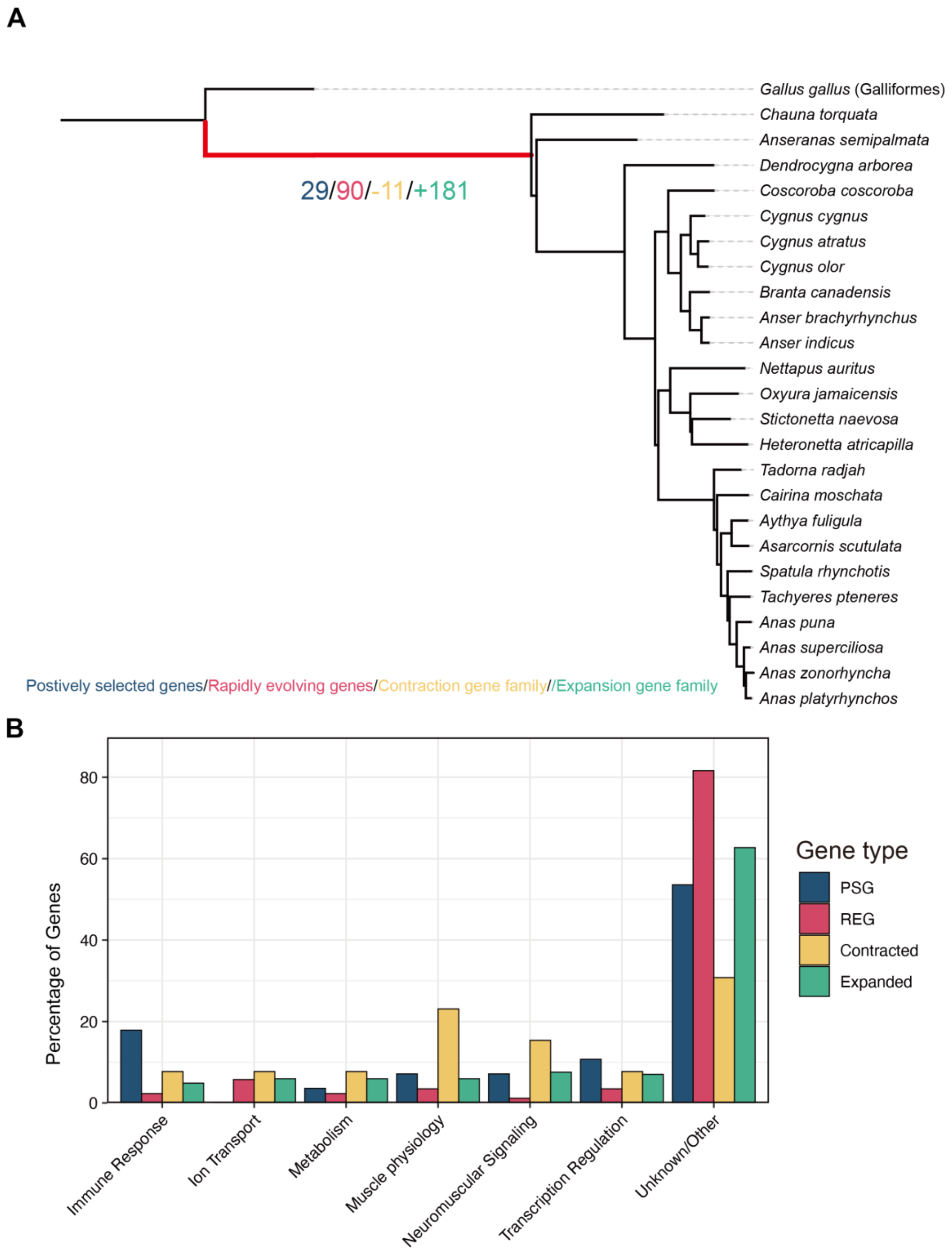

Figure S19. Positively selected genes (PSGs), rapidly evolving genes (REGs), expanded and contracted gene family are shown along the phylogenetic tree and GO terms enrichment of genes. (A) PSGs, REGs, and expanded and contracted gene family count at the base of extant waterfowl. The oblique line is a simplification of the branch length. (B) GO terms enrichment of different types of gene sets. Items with Fisher's exact test FDR value < 0.05 are shown. The bar graph shows the proportion of the number of genes in each GO term in different gene sets.

Table S1. Genomes used in this study and assembly numbers.

Species	Source
Gallus gallus	GCA_000002315.5
Chauna torquata	B10K-DU-011-36
Anseranas semipalmata	GCA_013399115.1
Dendrocygna arborea	This study
Coscoroba coscoroba	This study
Branta canadensis	GCA_002592135.1
Anser brachyrhynchus	GCA_964034855.1
Anser indicus	GCA_025583725.1
Cygnus cygnus	GCA_014362685.1
Cygnus olor	GCA_009769625.2
Cygnus atratus	GCA_013377495.2
Nettapus auratus	GCA_011076525.1
Oxyura jamaicensis	GCA_011077185.1
Heteronetta atricapilla	GCA_011075105.1
Stictonetta naevosa	GCA_011074415.1
Tadorna radjah	This study
Cairina moschata	GCA_018104995.1
Asarcornis scutulata	GCA_013398475.1
Aythya fuligula	GCA_009819795.1
Tachyeres pteneres	This study
Anas (Spatula) rhynchotis	This study
Anas puna	This study
Anas superciliosa superciliosa	This study
Anas platyrhynchos	GCA_002743455.1
Anas zonorhyncha	GCA_002224875.1

Table S2. The PSGs at the ancestral branch of waterfowl. PSGs are identified using PAML with branch-site models, and the corrected Chi-square test adjusted *p*-value cutoff is 0.05 after FDR correction.

Gene	Gene Symbol	Lnl0	Lnl1	*p*-value	Adjusted *p*-value
ENSGALG00010001183	NDUFA12	-1836.6	-1832.79	0.005812	0.092410219
ENSGALG00010001577	EXOSC2	-1244.41	-1239.98	0.002925	0.071542595
ENSGALG00010001695	RARS	-3277.32	-3281.35	0.004525	0.087749491
ENSGALG00010005311	GCH1	-2405.21	-2394	2.19E-06	0.000695481
ENSGALG00010005362	CCL20	-1800.58	-1796.99	0.007341	0.10032302
ENSGALG00010007857	SKP2	-307.678	-302.373	0.001124	0.051057837
ENSGALG00010007972	YME1L1	-4119.04	-4115.3	0.006278	0.095065532
ENSGALG00010009486	B4GALT1	-2129.52	-2123.06	0.000324	0.025781403
ENSGALG00010009654	WNT8A	-539.692	-535.521	0.003875	0.087749491
ENSGALG00010012250	CHMP6	-847.521	-844.639	0.016361	0.179411255
ENSGALG00010013290	SMARCB1	-963.677	-959.785	0.005273	0.088257155
ENSGALG00010013533	ATP6AP2	-2194.71	-2188.68	0.000514	0.030947174
ENSGALG00010013704	RPS3A	-662.046	-657.375	0.00224	0.068499515
ENSGALG00010014816	EXOC8	-2208.71	-2204.69	0.004573	0.087749491
ENSGALG00010015558	LOC419112	-3414.75	-3410.16	0.002437	0.068499515
ENSGALG00010016724	ATP1B1	-2530.97	-2525.9	0.001448	0.057539937
ENSGALG00010016931	ZPBP2	-1870.97	-1866.43	0.002585	0.068499515
ENSGALG00010022400	RARRES1	-3675.69	-3672.14	0.00766	0.10032302
ENSGALG00010023707	TST	-1618.63	-1614.68	0.004909	0.087749491
ENSGALG00010023872	NKX6-2	-671.543	-668.13	0.008982	0.10597287
ENSGALG00010024185	PGD	-2916.32	-2912.37	0.004967	0.087749491
ENSGALG00010024591	NELFB	-3666.66	-3660.74	0.000584	0.030947174
ENSGALG00010025971	UFL1	-2423.96	-2420.55	0.008998	0.10597287
ENSGALG00010025998	ATIC	-2513.79	-2510.51	0.0104	0.118109637
ENSGALG00010026951	KHDRBS1	-2133.74	-2129.05	0.002203	0.068499515
ENSGALG00010027568	NHLRC2	-4500.39	-4490.3	7.09E-06	0.001126577
ENSGALG00010027597	LMOD2	-3533.87	-3525.67	5.17E-05	0.005475588
ENSGALG00010029710	BLMH	-2722.92	-2719.26	0.006825	0.098645828
NA	ATP4B	-826.998	-823.469	0.007887	0.10032302

Table S3. The REGs at the ancestral branch of waterfowl. PSGs are identified using PAML with branch-site models, and the corrected Chi-square test adjusted *p* value cutoff is 0.05 after FDR correction.

Gene	Gene Symbol	Lnl0	Lnl1	*p*-value	Adjusted *p*-value
ENSGALG00010000303	PES1	-2382.04	-2356.37	7.79E-13	8.86E-11
ENSGALG00010001505	SSR3	-1067.22	-1057.35	8.82E-06	0.000216569
ENSGALG00010002497	NA	-982.232	-973.743	3.78E-05	0.000788935
ENSGALG00010003717	LEPROTL1	-506.629	-497.164	1.36E-05	0.000326523
ENSGALG00010003813	LRIT3	-2430.14	-2414.74	2.87E-08	1.20E-06
ENSGALG00010003995	MLNR	-1632.07	-1625.46	0.000276	0.004805071
ENSGALG00010005551	METTL14	-1537.43	-1531.97	0.00095	0.015059311
ENSGALG00010006283	DNAJC25	-1482.17	-1453.15	2.56E-14	3.57E-12
ENSGALG00010006336	ALDH8A1	-3068.53	-3058.46	7.18E-06	0.00018351
ENSGALG00010006961	ZCCHC14	-2232.18	-2223.66	3.67E-05	0.000778432
ENSGALG00010007485	CTSC	-1790.97	-1784.85	0.000467	0.007693117
ENSGALG00010008039	CHMP1A	-1154.38	-1147.37	0.000182	0.003342935
ENSGALG00010009114	SETD7	-2439.19	-2427.96	2.15E-06	6.00E-05
ENSGALG00010009672	DDX47	-2078.45	-2065.2	2.63E-07	9.68E-06
ENSGALG00010010262	HSPA9	-3757.2	-3738.15	6.74E-10	3.12E-08
ENSGALG00010010303	ETF1	-2330.42	-2311.25	5.96E-10	2.87E-08
ENSGALG00010010728	ELOVL2	-1202.29	-1194.65	9.31E-05	0.001792339
ENSGALG00010011251	FRZB	-810.383	-802.942	0.000114	0.00217101
ENSGALG00010011539	LANCL1	-1514.65	-1503.17	1.65E-06	4.70E-05
ENSGALG00010011992	YEATS4	-1591.34	-1584.44	0.000204	0.003640416
ENSGALG00010012083	MGP	-997.575	-992.759	0.001911	0.027507225
ENSGALG00010012756	RPA2	-1950.75	-1946	0.002052	0.029201264
ENSGALG00010012778	TBC1D15	-1064.08	-1055.96	5.61E-05	0.0011325
ENSGALG00010012883	DCAF13	-2439.1	-2434.09	0.001552	0.02286004
ENSGALG00010012887	KCNJ4	-3427.59	-3421.33	0.0004	0.006766283
ENSGALG00010012908	STRAP	-1129.12	-1109.62	4.25E-10	2.31E-08
ENSGALG00010012922	SNRNP40	-1067.01	-1061.93	0.001445	0.021543137
ENSGALG00010013353	NDFIP1	-1041.12	-1036.22	0.001732	0.025216083
ENSGALG00010013491	CNOT11	-1408.8	-1384.48	3.08E-12	2.97E-10
ENSGALG00010013562	RERGL	-1294.55	-1280.36	9.92E-08	4.01E-06
ENSGALG00010014083	NIPSNAP3A	-1211	-1204.84	0.000446	0.007453258
ENSGALG00010014482	THOC3	-1331.53	-1307.47	3.99E-12	3.57E-10
ENSGALG00010014519	BRS3	-2296.25	-2289.29	0.000191	0.003469355
ENSGALG00010014658	SELENOP1	-1258.17	-1252.93	0.0012	0.018327708
ENSGALG00010014682	IL13RA1	-3038.63	-3030.03	3.37E-05	0.000739886
ENSGALG00010015803	SYF2	-1712.64	-1704.55	5.73E-05	0.001137807
ENSGALG00010016480	GPX3	-729.285	-723.566	0.000719	0.011548176
ENSGALG00010016647	EGR1	-3238.08	-3230.95	0.00016	0.002983505
ENSGALG00010016777	TNMD	-1510.98	-1500.54	4.90E-06	0.000130622
ENSGALG00010017759	NODAL	-2328.33	-2319.14	1.82E-05	0.000422428

ENSGALG00010017821	BMP8A	-2586.31	-2572.63	1.69E-07	6.41E-06
ENSGALG00010018035	ITM2A	-2127.87	-2116.09	1.21E-06	3.70E-05
ENSGALG00010018430	LOXL4	-6736.44	-6684.15	0	0
ENSGALG00010018623	ARRDC4	-2125.12	-2112.4	4.57E-07	1.59E-05
ENSGALG00010019173	RHO	-2252.43	-2229.02	7.73E-12	6.05E-10
ENSGALG00010019803	FAH	-2951.88	-2917.49	1.11E-16	1.99E-14
ENSGALG00010019925	RDH11	-1219.45	-1211.31	5.51E-05	0.00113133
ENSGALG00010020055	R3HCC1	-2028.85	-2019.58	1.65E-05	0.000390644
ENSGALG00010020088	LMOD3	-2248.46	-2244.15	0.003314	0.046618125
ENSGALG00010020562	LRRN1	-1959.26	-1921.06	0	0
ENSGALG00010021541	CYP27A1	-1842.05	-1830.98	2.52E-06	6.87E-05
ENSGALG00010021727	MVK	-2723.4	-2718.25	0.001329	0.020047161
ENSGALG00010022257	NA	-2679.38	-2673.37	0.000527	0.008566301
ENSGALG00010022317	PSME3	-1081.7	-1056.34	1.06E-12	1.11E-10
ENSGALG00010022392	EMILIN3	-3886.69	-3881.34	0.00107	0.016540267
ENSGALG00010022545	CSTF3	-2573.9	-2516.77	0	0
ENSGALG00010022587	NA	-3414.51	-3395.11	4.70E-10	2.45E-08
ENSGALG00010022727	ACP2	-1327.26	-1315.09	8.11E-07	2.67E-05
ENSGALG00010023059	SELENOF	-401.641	-395.335	0.000383	0.006567824
ENSGALG00010023167	GPD1	-1817.87	-1806.29	1.48E-06	4.32E-05
ENSGALG00010023216	NA	-2237.19	-2228.43	2.82E-05	0.000631018
ENSGALG00010023454	HOXB1	-2274.61	-2262.79	1.17E-06	3.67E-05
ENSGALG00010023597	MOGAT1	-1010.23	-1001.66	3.48E-05	0.000750966
ENSGALG00010023714	EIF3M	-2609.39	-2597.39	9.65E-07	3.10E-05
ENSGALG00010023760	ZW10	-5529.84	-5519.46	5.19E-06	0.000135318
ENSGALG00010023930	SRPRA	-4704.5	-4660.95	0	0
ENSGALG00010024624	CTSD	-2002.5	-1974.21	5.37E-14	6.73E-12
ENSGALG00010024916	TMIE	-636.12	-612.641	7.25E-12	6.05E-10
ENSGALG00010025211	RAG1	-4952.06	-4931.78	1.91E-10	1.14E-08
ENSGALG00010025379	ACAD8	-2597.65	-2583.97	1.69E-07	6.41E-06
ENSGALG00010025549	DHCR24	-2799.95	-2790.97	2.25E-05	0.00051172
ENSGALG00010025855	CPO	-3132.59	-3122.65	8.27E-06	0.000207146
ENSGALG00010025948	WNT9B	-1525.53	-1505.84	3.50E-10	1.99E-08
ENSGALG00010026061	NA	-4352.09	-4330.74	6.36E-11	4.19E-09
ENSGALG00010026091	DTX4	-2354.15	-2322.01	1.11E-15	1.74E-13
ENSGALG00010027136	MAPKAPK2	-2160.85	-2137.5	8.30E-12	6.11E-10
ENSGALG00010027286	NA	-2500.34	-2479.88	1.60E-10	9.99E-09
ENSGALG00010028174	GMPPB	-2374.32	-2361.27	3.23E-07	1.16E-05
ENSGALG00010028206	RBM15B	-4934.75	-4864.02	0	0
ENSGALG00010029203	ALDOC	-1648.19	-1614	1.11E-16	1.99E-14
ENSGALG00010029219	TLCD1	-1647.77	-1636.14	1.41E-06	4.21E-05
ENSGALG00010029392	RTN4RL1	-3069.27	-3065.01	0.003499	0.048671374
ENSGALG00010029496	TTYH1	-4154.89	-4142.35	5.46E-07	1.85E-05
ENSGALG00010029502	BTBD17	-2952.01	-2944.16	7.43E-05	0.001453537

ENSGALG00010029627	PEX12	-2472.4	-2453.22	5.91E-10	2.87E-08
ENSGALG00010029632	TMEM132E	-6601.52	-6580.06	5.73E-11	3.98E-09
ENSGALG00010029694	ARMC7	-1460.71	-1442.55	1.68E-09	7.24E-08
ENSGALG00010029772	VPS53	-4426.4	-4407.42	7.23E-10	3.23E-08
ENSGALG00010029775	TRIM47	-3830.64	-3825.22	0.000999	0.015630117
NA	MC4F3	-1315.59	-1308.91	0.000257	0.004529313

Table S4. GO enrichment of REGs at the ancestral branch of waterfowl. REGs at the ancestral branch of waterfowl are input for GO enrichment analysis using WebGestalt. Items with Fisher's exact test FDR value < 0.05 are listed.

Category	Term	Background number	Input number	FDR-value
Biological_Process	GO:0043434~response to peptide hormone	67	3	0.010401188
Biological_Process	GO:0090090~negative regulation of canonical Wnt signaling pathway	25	2	0.012255194
Biological_Process	GO:0120254~olefinic compound metabolic process	25	2	0.012255194
Biological_Process	GO:0007167~enzyme-linked receptor protein signaling pathway	206	5	0.012493941
Cellular_Component	GO:0005789~endoplasmic reticulum membrane	206	5	0.012493941
Molecular_Function	GO:0016746~acyltransferase activity	134	4	0.012664867
Cellular_Component	GO:0098827~endoplasmic reticulum subcompartment	207	5	0.012738336
Cellular_Component	GO:0042175~nuclear outer membrane-endoplasmic reticulum membrane network	209	5	0.013236869
Biological_Process	GO:1901652~response to peptide	77	3	0.015147441
Molecular_Function	GO:0005125~cytokine activity	80	3	0.016773462

Table S5. Contracted gene families at the ancestral branch of waterfowl. The gene symbol indicates a representative gene of this gene family (Orthologues) in chicken.

Gene Symbol	Orthologues
F-KER	OG0000026
NFASC	OG0000201
MBNL1	OG0000224
RALGAPA1	OG0000228
PROM1	OG0000376
MBNL2	OG0000540
MHCY1	OG0000557
MBNL3	OG0000792
MHM2	OG0001236
PCDHA6	OG0001342
SLC31A1	OG0002427

Table S6. Expanded gene families at the ancestral branch of waterfowl. The gene symbol indicates a representative gene of this gene family (Orthologues) in chicken.

Gene Symbol	Orthologues
PCDH10	OG0000004
MYH7	OG0000006
DYRK2	OG0000008
B3GNT2	OG0000009
BTN1A1	OG0000010
BG1	OG0000011
PIT54	OG0000012
CD163L	OG0000014
OLFR4	OG0000015
OR8U1	OG0000017
OR1052	OG0000018
COR1	OG0000025
OLFR6	OG0000028
OR6B1	OG0000029
KRT7	OG0000034
ELAVL4	OG0000035
TCF12	OG0000038
EBF1	OG0000039
EBF2	OG0000040
NRCAM	OG0000041
CDH20	OG0000044
RYR3	OG0000045
TLE4	OG0000046
GRIA4	OG0000048
FAT3	OG0000055
TENM3	OG0000056
SCN9A	OG0000058
RBMS1	OG0000066
LOC431317	OG0000069
LOC431316	OG0000081
LOC100859586	OG0000083
LOC100859616	OG0000086
LOC425362	OG0000087
LOC100859722	OG0000090
LOC100859756	OG0000091
LOC396480	OG0000094
LOC396479	OG0000095
LOC426218	OG0000096
LOC395095	OG0000100
LOC100858504	OG0000102
LOC771066	OG0000107

TUBAL3	OG0000111
OR52R1	OG0000115
ADGRL2	OG0000118
NAV3	OG0000119
COL4A1	OG0000121
NBEA	OG0000122
GALNT18	OG0000132
ITPR1	OG0000137
TCF7L2	OG0000148
IQGAP2	OG0000149
EXOC6	OG0000154
PAK1	OG0000156
PDE4B	OG0000157
PICALML	OG0000158
HNRNPA1	OG0000161
PRSS2	OG0000162
RALGPS2	OG0000164
TRIM7.1	OG0000167
DMD	OG0000171
PPP3CB	OG0000174
MYH10	OG0000180
MEIS1	OG0000182
MTMR3	OG0000184
MTSS1	OG0000196
GRK4	OG0000202
COL5A1	OG0000204
ESRRG	OG0000211
RUNX1	OG0000213
KCNA4	OG0000219
GRB10	OG0000222
HOXA3	OG0000226
ATP2B1	OG0000227
TRIM2	OG0000230
CACNB4	OG0000234
CACNB4	OG0000235
KCNH6	OG0000238
CACNA2D1	OG0000242
H3C37	OG0000250
H3-3A	OG0000251
RALYL	OG0000262
FBXW11	OG0000273
KCNIP4	OG0000275
SRGAP1	OG0000279
SRGAP3	OG0000281

LRP1	OG0000303
KCNT1	OG0000311
DIAPH2	OG0000316
LOXL2	OG0000322
ANO3	OG0000324
GPR83	OG0000325
PTPRU	OG0000327
SERPINC1	OG0000332
LIMS1	OG0000339
PPP2R2D	OG0000349
PTP4A1	OG0000360
MAPK10	OG0000361
SLC35A3	OG0000373
SBF2	OG0000378
ENOX2	OG0000384
LOC396380	OG0000387
GSTA4	OG0000388
H2BC38	OG0000398
PTPRA	OG0000405
OLFM1	OG0000407
MPPED2	OG0000428
MTA1	OG0000440
ABCA1	OG0000449
NEU3	OG0000453
CCR5	OG0000465
CTNNA3	OG0000471
CDC42BPA	OG0000472
MYO1A	OG0000473
ZFHX3	OG0000479
SLC25A13	OG0000485
PPM1B	OG0000489
ANK3	OG0000491
CPNE8	OG0000498
LAMP3	OG0000501
FSTL4	OG0000503
FYN	OG0000513
FGR	OG0000521
HERC3	OG0000551
ASIC1	OG0000577
PCDH15	OG0000620
SIRPA	OG0000633
DGKZ	OG0000638
GNB1	OG0000655
CCSER2	OG0000717

SMARCA2	OG0000750
CBL	OG0000769
DYM	OG0000778
TLL1	OG0000872
H2AC38	OG0000883
-	OG0000931
GRM5	OG0000972
SEC23B	OG0000979
KCNAB1	OG0001031
NTRK3	OG0001081
PRKCD	OG0001090
MID1	OG0001091
TCTN3	OG0001092
GBP	OG0001093
SEPTIN5	OG0001094
FBXW7	OG0001106
PXN	OG0001131
SSBP3	OG0001164
FLOT2	OG0001167
SPTBN1	OG0001175
TULP1	OG0001176
NR2F2	OG0001177
TNS3	OG0001195
TPST2	OG0001213
LOC408038	OG0001215
ORMDL2	OG0001293
ZDHHC18	OG0001378
PPP2R5C	OG0001484
EPB42	OG0001887
NIPA2	OG0001922
PHF20L1	OG0001923
VSNL1	OG0001924
IL21R	OG0001925
MGAT4F	OG0002232
NKIRAS2	OG0002325
PCDH1	OG0002551
SPINK5	OG0002362
ZBTB7A	OG0002509
CTDSPL	OG0002408
POR	OG0002811
CYP4V2	OG0002513
PAPOLB	OG0002494
CDC42SE2	OG0002706
CSTB	OG0002395

MYO6	OG0002522
CABP2	OG0002692
PGLYRP2	OG0002646
PLAG1	OG0002549
JMJD1C	OG0002804
IGLL1	OG0002938
P2RY6	OG0003773
FAM46A	OG0003837
